# Are exposure to health information and media health literacy associated with fruit and vegetable consumption?

**DOI:** 10.1186/s12889-023-16474-1

**Published:** 2023-08-16

**Authors:** Nongnuch Jindarattanaporn, Jongjit Rittirong, Sirinya Phulkerd, Sasinee Thapsuwan, Natjera Thongcharoenchupong

**Affiliations:** Institute for Population and Social Research, Mahidol University, Nakorn Pathom, 73170 Thailand

**Keywords:** Health information, Media health literacy, Fruit and vegetable consumption, Youth

## Abstract

**Background:**

Health information promotes a healthy lifestyle; however, media health literacy (MHL) is essential to personal intake, especially fruit and vegetables (F&V). This study aimed to determine how exposure to health information and MHL affect F&V consumption among Thai youth aged 10–14 years. Health behavior at this age can be an important determinant of consumption habits when transitioning into adulthood.

**Methods:**

A stratified two-stage sample survey was conducted to recruit 1,871 youth across regions to be nationally representative. Qualtrics offline survey application was used for data collection face-to-face with all respondents. Multiple linear regression was used to analyze the explanatory variables on fruit consumption and vegetable consumption.

**Results:**

This study found that almost 70% of Thai youth were exposed to health-related content via the Internet, and had low analytical skills to process that information. Grade Point Average (GPA), exposure to health information, various media types, frequency of exposure to health information, and MHL influenced the frequency of F&V intake. Health status related to fruit intake, age, place of residence, amount of pocket money, and body mass index (BMI) were associated with F&V consumption.

**Conclusion:**

Exposure to health information and MHL are associated with F&V intake. Therefore, exposure to health information and MHL should be addressed for policy formulation in Thai schools and the health system.

**Supplementary Information:**

The online version contains supplementary material available at 10.1186/s12889-023-16474-1.

## Background

Globally, 1% (16 million) of disability-adjusted life years (DALYs) and 2.8% (1.7 million) of deaths are associated with insufficient fruit and vegetable (F&V) intake [1]. F&V consumption helps to reduce the risk of cardiovascular disease and cancer [2, 3]. Insufficient F&V intake among youth can impair a child’s caloric intake, and increases the risk of obesity and obesity-related illness [4, 5]. In addition, low F&V consumption is associated with lower academic achievement in childhood [6].

Worldwide, the average daily intake of F&V is estimated to be 20 to 50% short of the recommended level of at least 400 grams (g), or five servings per day [7, 8]. In Thailand, 25% of youth aged 6–14 years ate vegetables every day, and 16.2% % of youth aged 6–14 years ate fruit every day [9], and their daily F&V consumption was 201 g, and that is well below the recommendation for this age group (320 g) [10]. Thailand has a program, which is Thai School Lunch, to promote quality schools’ lunches. This program helps school estimate their raw material costs and calculate the nutrients of food recipes. The program is linked to farmers' databases in each area. Enabling farmers to manage their produce and supply to the school [11, 12].

While Thailand has a school lunch program to increase F&V consumption and promotes childhood nutrition, Thai youth still have insufficient F&V intake [12]. The proportion of F&V in daily food consumption among Thais aged 6–14 years decreased from 41% in 2013 to 23% in 2017 [13, 14]. By contrast, Thai youth consume more food high in fat, sugar, and salt today than ever. The percentage of foods that are high in sugar, salt, and fat consumed by Thai youth increased from 41% in 2013 to 71% in 2017. Consumption of sugar-sweetened beverages rose from 90 to 93%; consumption of snacks that were high in fat increased from 89 to 93%; and consumption of “fast food” increased from 44 to 46% during the same period [13, 14].

There are many factors that influence F&V intake among youth, such as gender, age, socio-economic status of the child’s family, youth attitudes toward F&V, availability of F&V at home, and the socio-demographic characteristics of parents [10, 15, 16]. In addition, determinants of improved F&V consumption among youth include exposure to positive health-related content, media literacy (ML), and health literacy (HL) [17–19]. Media such as the Internet and television are significant sources of health information [20, 21], and exposure to health messages can influence youth to increase their F&V intake [22]. In Thailand, factors associated with ML and HL include gender, age, education, stress, and communication skill [23]. Therefore, ML and HL play a major role in shaping and reinforcing attitudes and healthy eating in youth, and these habits can extend into adulthood [24–26].

Complementarity of ML and HL is needed because it empowers youth to protect and promote their health throughout their life [18]. Hence, youth can make appropriate health behavior decisions when using media [27, 28]. In the past, ML and HL in the literature were investigated independently [28]. By contrast, a study in Israel merged the concepts of ML and HL as ‘*MHL*,’ which is considered to be an indicator of the ability to evaluate health content through media [20]. MHL can help to illuminate the analytical and critical processing of health information through media by youth [20]. Individuals between the ages of 10 and 14, in particular, are in a crucial stage of their development where they begin to form their health literacy through exposure to media [29, 30]. Making informed decisions regarding their diet during this period can have a lasting impact on their future well-being and adopting a healthy lifestyle into adulthood [29, 30]. By examining the association between health information exposure and MHL on food consumption, we can better understand this population and effectively improve health interventions to address their needs. Although the synergy of ML and HL is addressed, there is no data regarding MHL among Thai youth and the association between exposure to health-related content, MHL, and F&V intake in Thailand.

The level of media health literacy (MHL) among youth is influenced by various environmental factors. To understand this phenomenon, we employ the Socio-Ecological Model (SEM), which takes into account individual, interpersonal, community, and societal factors and their interactions that shape people's behavior [31, 32]. Previous studies have provided support for the concepts of SEM and MHL, indicating that socio-demographic characteristics and exposure to health information associated with MHL can impact healthy behaviors in adolescents [20]. Considering that individual health status plays an important role in shaping adolescent behavior [33], it is crucial to analyze its influence on health information exposure and MHL [23]. Furthermore, we incorporate fruit and vegetable consumption into our research model to assess its impact on MHL. Consequently, this study aims to investigate the relationship between health information exposure, MHL, and fruit and vegetable consumption among Thai individuals aged 10–14 years. Hence, this study examined the influence of exposure to health information and MHL on F&V consumption among Thais aged 10–14.

## Methods

A national, cross-sectional study of Thais aged 10–14 was conducted from March to September 2020.

### Sampling and sample size

Based on data provided by the Thailand National Statistical Office (NSO), approximately four million Thai individuals aged 10–14 were surveyed in 2017 [29]. To ensure a representative sample at the national level, a multi-stage stratified sampling design conducted by the NSO.

Initially, a random sampling method was employed to select eight provinces from four regions (North, Northeast, Central, South) and Bangkok (the capital city of Thailand). Subsequently, two provinces were randomly chosen from each region. Secondly, all population within each province was divided by all enumeration areas (EAs) of each province. From this calculation, there were 100 EAs across nine provinces, comprising 55 urban EAs and 45 rural EAs [29], with 2,500 participants of Thai residents aged 10–14 years. Thirdly, systematic sampling was utilized to minimize selection bias when selecting the EAs. The list of EAs was generated by geographic distribution of nine selected provinces and all regions across countries. The number of households that responded in each EA ranged from 20 to 25. Fourthly, within each EA, every household on the list was approached to participate in the study. A total of 25 respondents were expected from each EA. All Thai residents aged 10–14 who fulfilled the inclusion criteria were interviewed at their homes.

**Fig. 1:**
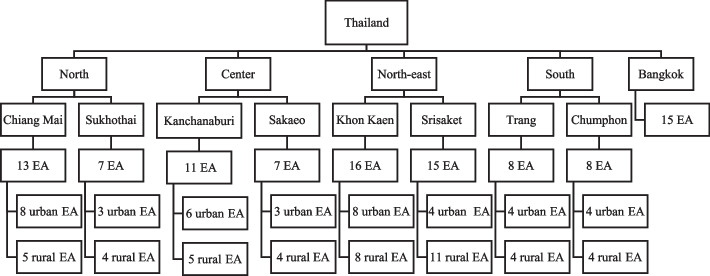
Sampling Frame

In cases where the data collector could not reach a selected resident after two visits to the household, that child was excluded from the study, and no replacement was sought. Out of the intended sample size of 2,500 participants across 100 EAs, 1,871 individuals agreed to be interviewed, resulting in a response rate of approximately 75%. The sampling frame is depicted in Fig. 1.

### Instrument

Based on a review of related literature in many countries, including Thailand, the questionnaire was designed to include the following five parts: Socio-demographic characteristics, media exposure, exposure to health information, MHL, and consumption of F&V. The development of the questionnaire used the explanatory-sequential approach [32], consisting of the following steps: [1] Documentary research to review the concept of media exposure, exposure to health information, MHL, and consumption of F&V and to develop and design questionnaires; [2] Purposive sampling was used to select key informants (KIs) and children. Interviews with eight KIs who have more than five years of experience (two public health experts, one marketing communication expert, one advertising expert, two youth media experts, and two child and adolescent psychology experts) in order to review structure and content of the questionnaires, and focus group discussions with six youth age 10–14 years who were similar to our samples in order to select the popular video clips; [3] Review of the draft questionnaire by five experts (public health, marketing communication, advertising, media for youth, child and adolescent psychology) in order to assess the content validity ratio (CVR) and content validity index (CVI); and [4] Pre-testing the revised draft instrument with 40 youth age 10–14 year to test the reliability of the questionnaire [33].

To evaluate MHL, the researchers conducted two focus group sessions with Thai youths aged 10–14 years to identify popular video clips in their age group [34]. Findings from the focus group revealed that the most popular video clips among Thai children were the salty snack video, the fried chicken video, the Eating 2:1:1 video, and the Let's start running video again. Then, the four video clips were selected and used in the questionnaires [34]. Regarding the discussion, we selected two clips that contained information about the consumption of a healthy diet, and two videos were about unhealthy diet intake: (1) an advertisement for salty snacks-potato chips (2) an advertisement for fast food-fried chicken (3) healthy eating behavior- “Eating 2:1:1” of veggie, rice, and meat respectively [35], and [4] encouraging for exercise- “Let's start running again…win in defeat”. The trained researcher asked all respondents to watch them and then ask MHL questions.

The researchers asked the youth to carefully watch the videos and then respond to the same 15 questions for each clip (see Supplementary material file #1). For content validity, the CVR and CVI evaluation form was adapted from Zamanzadeh V, et al., 2015 [35] and sent to five experts. The experts rated each question on relevance and clarity in order to calculate the CVI and CVR. CVI and CVR for all items were computed from measurements on a 4-point ordinal scale: for CVI, “1” means did not clear, “2” refers to an item that needs some correction, “3” means clear but needs slight correction, “4” refers to highly clear. CVR, “1” refers to did not relevant, “2” means slightly relevant, “3” refers to fairly relevant, and “4” means very relevant. Each question of the CVI was evaluated by the proportion of experts who scored each item with a “3” or a “4” [35]. The CVI and CVR of each item were calculated according to scores; if CVI and CVR are higher than 0.75, the items will be appropriate. If they are between 0.70–0.75, they need revision. If they are less than 0.70, they are eliminated [35]. The CVR and CVI of the four clips were 0.768 and 0.884 for healthy and unhealthy diet content, respectively [33]. For reliability, the reliability test was employed by a self-administered questionnaire on the 40 students two weeks apart. The criteria of reliability defined 0.4 to 0.59 as fair, 0.60 to 0.74 as good, and above 0.75 as excellent [36]. The test-reliability of the MHL questionnaire based on the Pearson correlation coefficient between the two sets of scores for video clip # 1 was *r* = 0.80, 2 (*r* = 0.73), 3 (*r* = 0.69) and 4 (*r* = 0.76) [33]. Before data collection, a hands-on training workshop was organized with 12 interviewers. The workshop aimed to help them understand the study protocol, questionnaire, and assigned tasks and develop hands-on survey skills. The researchers and well-trained staff used Qualtrics offline survey application to administer the survey on a mobile device (e.g., tablet computer, smartphone) without the Internet. The data were collected by face-to-face interviews, with all respondents using the structured questionnaire. A research team contacted the village head to help coordinate with the targeted households and bring the team to meet the child from each household. Before each interview, parents or guardians were asked for permission (to sign the consent) to interview their child. Data were collected from March to September 2020.

## Variables

### Dependent variable

#### F&V consumption

This variable was measured by the following question: "*Last week, how often have you eaten the following food: (1) vegetables and (2) fruit?*" The response was the average frequency of vegetable and fruit consumption (i.e., days per week). Therefore, F&V consumption of the participants was collected based on the frequency (number of days) that the participant consumed F&V in a typical week. These two variables were continuous.

### Independent variables

#### Exposure to health information

This section consisted of four parts. The first part measured the use of media using the following yes/no question: "*In the last week, have you seen any eating and exercise content in any of the following media: Internet, television, mobile phone text messages (SMS), personal media (parents or guardians, relatives, siblings, teachers, friends), and/or books/textbooks*?" The second part measured types of media used to access health information in the past week. Then the number of channels of media types accessed with health information last week was computed on a composite scale that combined yes answers in the type of media variable. The third part asked about the frequency of exposure to health-related content through media. The response was coded as the average frequency of exposure to health information, such as three times per day (times per day). The fourth part asked about the duration of exposure to health information, such as 30 min per time (minutes per exposure). Therefore, these three variables were continuous.

### MHL

The following four ability domains comprise MHL: Perceiving and understanding, analysis, evaluation, and intent to act. Four domains were measured using four video clips and 15 questions for each clip video, in which a correct answer was scored = 1 and an incorrect answer = 0. Then, MHL was computed on a composite scale that combined the four domains for each clip video. The total score for each clip was 15, and the total score for all four video clips was 60. Hence, the MHL variable was continuous.

### Confounding factor

#### Socio-demographic characteristics

This section recorded the study subject's gender, age as a continuous variable, place of residence (urban/rural), GPA as a continuous variable, pocket money (average per day) as a continuous variable, and health status (has/hasn’t a chronic illness). To calculate body mass index (BMI) as a continuous variable, the field data collection teams measured the participants’ weight and height on site. The weight measurement used a digital scale placed on a firm surface. Participants took off their shoes and stood with both feet in the middle of the scale. For height, the participants stood with their backs against the wall with their feet together, and a tape measure was used to record height. The researchers consider the International Obesity Taskforce (IOTF) guidelines to establish a cutoff for BMI groups in order to describe the obesity status.

#### Data analysis

The socio-demographic status of the family of participating youth, exposure to health information, MHL, and F&V consumption were processed as frequencies and percentages. Correlations and multi-collinearity of the variables were examined before conducting regression analysis. Multiple linear regression was used to determine the relationship between independent variables and F&V consumption. The enter, which is the default method for the multiple linear regression analysis, was conducted to select all independent variables to include in a single model because all independent variables were given equal importance in the analysis [37]. A *p*-value < 0.05 was considered statistically significant. The linear relationship was evaluated between the dependent variable (F&V consumption) and independent variables. In addition, the normal distribution of the independent and dependent variables, ANOVA or model fit testing, t-test for testing of association between independent and dependent variables, VIF for collinearity testing, Tolerance for collinearity testing, and error were tested to confirm regression assumptions(see results in Tables 5 and 6). The criteria of these statistics were set as follows: ANOVA and t-test < 0.05, VIF < 1, Tolerance near 1.0, error = 0, normal distribution, independence of error, and the variance of error of x = ơ^2^ or Durbin Watson between 1.5–2.5 [38]. SPSS version 22 was used to analyze the frequency distributions and statistical relationships of the variables.

## Results

### Socio-demographic characteristics of the sample

Of the total sample of 1,871, 55.3% were male, and 44.7% were female. The mean (± standard deviation, sd) age was 11.9 (± 1.4) years and ranged from 10 to 14. About two-fifths had a GPA in the range of 3.00–3.50 (41.8%). Half the respondents (50.5%) lived in rural areas. Almost three-fifths of the sample had pocket money for buying sweets and beverages, ranging from 20–50 baht per day (approximately US$ 0.6 – 1.5). Over one-third had less than 20 baht allowance per day (37.8%), while a small minority had more than 50 baht (2.9%). Most youths had no chronic illness, and half (51.8%) were underweight. See Table 1.

**Table 1:** Socio-demographic characteristics of participants

Variables	All (*n* = 1,871)	
	**n**	**%**
Gender		
Female	1,035	55.3
Male	836	44.7
Age (years) (median = 12, mean = 11.9, sd = 1.353, max = 14, min = 10)		
10	336	18.0
11	405	21.6
12	455	24.4
13	371	19.8
14	304	16.2
Place of residence		
Rural	945	50.5
Urban	926	49.5
GPA (median = 3.10, mean = 3.10, sd = 0.665, max = 4, min = 1)		
Low (1.00–2.99)	586	31.3
Moderate (3.00–3.50)	781	41.8
High (3.51–4.00)	504	26.9
Amount of pocket money (baht per day) (median = 20, mean = 22.3, sd = 15.926, max = 120, min = 5)		
< 20	706	37.8
20–50	1,110	59.3
> 50	55	2.9
BMI (IOTF) (median = 18.17, mean = 19.3, sd = 4.409, max = 44.6, min = 10.7)		
Underweight	969	51.8
Normal	563	30.1
Overweight/obese	339	18.1
Health status		
Without a chronic illness	1,688	90.2
Having a chronic illness	183	9.8

*US$ 1 is approximately 35 baht (as of the end of 2022)*

### Exposure to health information

Almost 70% of youth were exposed to health-related content through the Internet, 45% were exposed to health information via media less than three times per day, and 83% were exposed to health information via media 10 min or less per time. The mean (± sd) number of media types used to access health information in the last week was 1.8 (± 0.8) sources with a range of 1 to 3 (Table 2).

**Table 2:** Exposure to health information among Thai youth

Variables	All (*n* = 1,871)	
	**n**	**%**
Type of media		
Internet	1,305	69.7
Television	472	25.2
Other (SMS, personnel media, print media such as books/textbooks)	88	4.8
Not exposed	6	0.3
The number of media types used to access health information in the past week (median = 2, mean = 1.8, sd = 0.797, max = 3, min = 1)		
1	918	49.1
2	578	30.9
3	375	20.0
Frequency of exposure to health information (median = 3, mean = 3.26, sd = 2.284, max = 10, min = 1)		
< 3 times per day	844	45.0
3–4	572	30.5
5–10	455	24.4
Duration of exposure to heath information (median = 3, mean = 9.0, sd = 12.747, max = 60, min = 0.15)		
≤ 10 min per time	1,553	82.9
11–20	162	8.6
21–30	99	5.3
31–60	57	3.2

### MHL Scores

MHL comprises four ability domains: Perceiving and understanding, analysis, evaluation, and intention to act. The total possible score for the MHL index is 60. In this study, the overall mean score of MHL was 36.8 (± 6.6), with a minimum of 12 and a maximum of 54. The median score for MHL was 37. The maximum potential scores for perceiving/understanding, analysis, evaluation, and intention to act are 11, 20, 12, and 16, respectively. The mean score for perceiving and understanding was 7.3 (± 1.7), ranging from 0 to 11. The mean score for analysis was 9.6 (± 3.2, min = 1 and max = 20). The mean score for evaluation was 9.2 (± 2.3, min = 0 and max = 12). The total mean score for intention to act was 10.5 (± 3.7, min = 0 and max = 16). See Table 3.

**Table 3:** Average MHL scores of Thai youth by domain and diagnostic questions

Variables	n	Median	Mean	sd	Min	Max
**Overall MHL score**	1,871	37	36.8	6.580	12	54
**1. Perceiving and understanding**	**1,871**	**8**	**7.3**	**1.736**	**0**	**11**
1.1 What product does this advertising sell?	1,871	3	2.5	0.909	0	4
1.2 What content has appeared in the media that you just watched to present a story about?	1,871	2	1.9	0.931	0	3
1.3 Is there content about health appearing in the media?	1,871	3	2.9	0.546	0	4
**2. Analysis**	**1,871**	**10**	**9.6**	**3.207**	**1**	**20**
2.1 What would be the purpose of this advertisement?	1,871	2	1.8	0.956	0	4
2.2 Who created this video clip?	1,871	1	1.2	1.237	0	4
2.3 Where do the content and information from the presenter or the presenter in the advertisement come from?	1,871	2	1.7	1.135	0	4
2.4 Who is the target audience?	1,871	3	2.5	0.967	0	4
2.5 Who benefits from this video clip?	1,871	2	2.4	1.007	0	4
**3. Evaluation**	**1,871**	**10**	**9.2**	**2.270**	**0**	**12**
3.1 Do you like this video clip?	1,871	4	3.2	1.009	0	4
3.2 Is this video clip reliable?	1,871	4	3.3	0.909	0	4
3.3 Is this video clip beneficial for you?	1,871	2	2.7	0.909	0	4
**4. Intention to act**	**1,871**	**11**	**10.5**	**3.716**	**0**	**16**
4.1 After watching this video clip, do you intend to do what it suggests?	1,871	3	2.7	1.118	0	4
4.2 Do you intend to tell your parents or guardians to do what it suggests?	1,871	3	2.6	1.191	0	4
4.3 Do you intend to tell your friends to do what it suggests?	1,871	3	2.6	1.246	0	4
4.4 Will you apply the information from this video clip in your daily life?	1,871	2	2.6	1.042	0	4

### Frequency of F&V consumption

Thai youth consumed vegetables on an average of 4.4 (± 2.5) days per week (median = 5) with a range of 0 to 7. They ate fruits 3.1 (± 2.2) days per week (median = 3) with a range from 0 to 7 (Table 4). Supplementary material file #2 presents disaggregated data on the consumption of F&V by socio-demographic characteristics of the sample.

**Table 4:** F&V intake among Thai youth (days per week)

Variables	Number of respondents	Median	Mean	Standard deviation	Minimum	Maximum
Eating (days per week)						
Vegetable	1,871	5	4.4	2.483	0	7
Fruit	1,871	3	3.1	2.247	0	7

### Association between exposure to health information, MHL, and frequency of fruit consumption

The analysis found that the number of media types accessed with health information in the previous week, frequency of exposure to health information, duration of exposure to health information, MHL, GPA, and health status were significant predictors of fruit consumption. The model indicates that youth who accessed more media types (β = 0.079), were exposed to health information more often (β = 0.061), and spent more time exposed to health information (β = 0.079) were more likely to have frequent fruit intake. Youth having a higher MHL (β = 0.085) and who had a higher GPA (β = 0.128) were more likely to consume fruit more often (Table 5).

**Table 5:** Multiple regression: fruit consumption as the dependent variable

Variables	b_i_	SE	β_i_	Sig	95% CI	
					**Lower Bound**	**Upper Bound**
(Constant)	-0.086	0.605		0.887	-1.273	1.101
Gender	-0.078	0.108	-0.017	0.471	-0.289	0.134
Age	0.045	0.039	0.027	0.250	-0.032	0.122
Place of residence	0.014	0.104	0.003	0.890	-0.189	0.217
GPA	0.433	0.082	0.128	0.000	0.273	0.594
Amount of pocket money	0.001	0.004	0.004	0.865	-0.006	0.008
BMI	-0.019	0.012	-0.038	0.101	-0.042	0.004
Health status	-0.359	0.171	-0.048	0.036	-0.695	-0.023
Number of media types accessed with health information last week	0.229	0.068	0.079	0.001	0.094	0.363
Frequency of exposure to health information	0.060	0.023	0.061	0.009	0.015	0.105
Duration of exposure to health information	0.005	0.001	0.079	0.001	0.002	0.008
MHL	0.029	0.008	0.085	0.000	0.013	0.045
Total R	0.245					
Total R^2^	0.060					
Total Adjusted R^2^	0.055					

*n* = 1,871, histogram was normal distribution curve, Residual mean = 0, F=10.186 (*p*<=0.000), VIF of each independent variable <0.87, Tolerance of each independent variable >1.0 , error using Durbin Watson =1.950

*SE* Standard error, *CI* Confidence interval

### Association between exposure to health information, MHL, and frequency of vegetable consumption

Socio-demographic characteristics of the sample, including age and sex, were controlled in the model, together with children’s GPA, amount of pocket money, BMI, and health status, as confounders, with all other variables held constantly in the model. The regression analysis of the frequency of vegetable consumption revealed that the number of media types accessed with health information in the previous week, frequency of exposure to health information, MHL, age, place of residence, GPA, amount of pocket money, and BMI were predictors of frequency of vegetable intake. The model identified that youth who had a higher number of media types accessed with health information in the previous week (β = 0.059) and who had a higher frequency of exposure to health information (β = 0.060) were more likely to have more frequent vegetable intake compared to those who had lower exposure to health information. In addition, youth with higher MHL scores (β = 0.101) tended to consume vegetables more often. By contrast, those having a larger amount of pocket money (β = -0.081) and living in an urban area (β = -0.064) were less likely to consume vegetables (Table 6).

**Table 6:** Multiple regression: vegetable consumption as the dependent variable

Variables	b_i_	SE	β_i_	Sig	95% CI	
					**Lower Bound**	**Upper Bound**
(Constant)	-1.042	0.672		0.121	-2.360	0.276
Gender	0.165	0.120	0.033	0.167	-0.069	0.400
Age	0.195	0.043	0.105	0.000	0.110	0.280
Place of residence	-0.318	0.115	-0.064	0.006	-0.544	-0.093
GPA	0.305	0.091	0.082	0.001	0.127	0.483
Amount of pocket money	-0.014	0.004	-0.081	0.001	-0.021	-0.006
BMI	0.033	0.013	0.059	0.011	0.008	0.058
Health status	-0.240	0.190	-0.029	0.207	-0.612	0.133
The number of media types accessed with health information last week	0.188	0.076	0.059	0.014	0.039	0.337
Frequency of exposure to health information	0.065	0.025	0.060	0.011	0.015	0.115
Duration of exposure to health information	-0.001	0.002	-0.020	0.387	-0.005	0.002
MHL	0.038	0.009	0.101	0.000	0.020	0.056
Total R	0.230					
Total R^2^	0.053					
Total Adjusted R^2^	0.047					

*n* =1,871, histogram was normal distribution curve, Residual mean = 0, F=9.416 (*p*<=0.000), VIF of each independent variable <0.87, Tolerance of each independent variable >1.1, error using Durbin Watson = 1.947

*SE* Standard error, *CI* Confidence interval

## Discussion

This study describes exposure to health information and MHL among Thai youth and examines factors related to F&V consumption. The majority of Thai youth in this sample were exposed to health-related content through the Internet, and the participants had a moderate level of MHL. When considering each element of MHL, Thai youth had limited capacity for analytical thinking. The test of the research hypothesis about F&V consumption found that exposure to health information and MHL were the most important predictors of F&V intake. Other factors, including GPA, and health status influenced fruit consumption; age, place of residence, GPA, amount of pocket money, and BMI were associated with vegetable consumption.

The MHL score of Thai youth was around 37. The researchers found that Thai youth had relatively low analytical scores when considering the MHL domains. This finding is consistent with the Program for International Student Assessment (PISA), which is periodically conducted in countries around the world. According to PISA data, Thai students had low literacy, where ‘literacy’ refers to a student's competence in applying knowledge and skills in core subjects to daily life, and having the ability to analyze, reason, and communicate effectively to identify the core content and interpret/assess it accurately [39]. Thus, MHL reflects a child’s competence, and literacy is related to teaching and learning methods that help to develop analytical and reasoning skills. Thailand and ASEAN neighboring countries (e.g., Malaysia, Indonesia, and the Philippines) employ teaching methods requiring minimal critical thinking skills [40–42]. Therefore, the PISA scores for these countries are relatively lower than Singapore and China, where active learning and problem-solving are more heavily practiced in school [43–46]. Therefore, an appropriate teaching and learning approach is essential for boosting MHL among Thai youth.

This study found an association between exposure to health information (types and frequency), and frequency of F&V consumption. This result is consistent with studies in nine European countries (Austria, Belgium, Denmark, Iceland, the Netherlands, Norway, Portugal, Spain, and Sweden). Those studies found that youth with a higher frequency of exposure to F&V information through various media (particularly TV, Internet, and print media) had higher F&V intake [47–49]. In addition, youth who are exposed to health-related content via media become inherently more familiar with the concepts and messages. This understanding informs the decision to consume F&V [47]. Moreover, the Thai study found that the higher the MHL score, the more likely that youth will consume F&V. The findings confirm that the concept and outcome measures relate to HL, potentially improving healthy eating behavior, or changing F&V consumption for the better [50]. In addition, the findings of this study correspond to similar studies in the USA, Taiwan, and Thailand in that HL was an essential factor influencing F&V intake frequency among youth [17, 51, 52]. However, the level of F&V consumption by Thai youth is still worrisome, as only 2–3 in 10 Thai youth eat enough F&V to meet the recommended amount [53]**.** That said, improved HL should boost F&V intake [51]. What is more, improved MHL should result in Thai youth eating more F&V more frequently.

Youth with a higher GPA had a greater frequency of F&V consumption than those with a lower GPA. This result is consistent with studies in Canada and Iceland which found that higher academic performance of high school students (grade 7–9) was associated with greater F&V consumption [54, 55]. This may be due to the probability that youth with a higher GPA is also superior in cognition, attentiveness, memory, extraction, and reasoning [56]. In addition, cognitive processes are the steps for using knowledge, combining knowledge with new knowledge, and making decisions based on that knowledge [57]. Therefore, youth with a higher GPA will be in a better position to understand, analyze, and evaluate health information which, in turn, may prompt them to consume F&V more than those with a lower GPA.

This study found a negative association between place of residence, pocket money, and vegetable consumption. A survey of purchasing behavior among primary grade 6 students in Thailand found that most students had 21–40 baht (US$ 0.60–1.20) a day for snacks and were more likely to buy unhealthy food and beverages with their allowance. Typically, Thai youth tended to purchase sweetened drinks (91.7%), snacks (67.9%), bakery goods (46.4%), and sweets (39.3) [58] in convenience stores, which are primarily located in urban areas. What is more, most convenience stores in Thailand do not carry fresh F&V [59]. This behavior can be seen among youth in China, Ghana, and the UK [15, 60, 61]. In this study, health status was associated with fruit consumption. Youth with chronic illness consumed more fruit than youth without illness, possibly because they were aware of their compromised health status and the need for good self-care behaviors [62, 63]. Moreover, parents and families are likely to be more concerned about a child’s health condition if s/he suffers from a chronic condition [64]. This supposition corresponds with the theory of family self-management of chronic disease in that the family is the primary healthcare provider. The individual or sufferer is involved in managing their health care, and family members are involved in caring for and supporting the ill person to maximize their quality of life and enable them to live in harmony with their family and peers [65, 66].

### Limitations

Although the sample is nationally-representative, the data collection was cross-sectional. Thus, it is impossible to conclude causality between the independent and dependent variables. Also, the survey did not collect the quantity (number of servings) of F&V consumed daily. Thus, this study can not report the sufficiency of F&V consumption according to recommendations by the World Health Organization (WHO) (at least five servings or 400 g per day) [67]. The study did not include other factors which are probably significantly related to F&V intakes, such as youth attitude toward F&V, F&V availability at home, and the socio-demographic characteristics of their parents [10, 15]. Hence, future studies should measure those variables and the amount of F&V consumed per serving or time, among other potential determinants and outcome variables.

## Conclusions

The results of this study emphasize that the Internet is a popular source of health information for today’s Thai youth. However, the critical thinking skills of Thai youth are low compared to other countries in Asia and the developed world in general. Accordingly, the Ministry of Education should improve the analytical ability of Thai youth by using active and thinking-based learning methods. Exposure to health information, MHL, and GPA of Thai youth was associated with their F&V intake. Therefore, schools and the public health sector should promote and disseminate F&V information through all popular channels with youth, particularly social media platforms and websites on the Internet. Thai youth with more pocket money less frequently consumed vegetables than those with less, and those with a chronic illness were more likely to consume fruit than those without a chronic illness. The campaigns for F&V consumption should be implemented in urban areas, especially among youth with pocket money and without a chronic illness.

### Supplementary Information

**Additional file 1.**

**Additional file 2.**

## Data Availability

The datasets used and/or analysed during the current study available from the corresponding author on reasonable request**.**
