# Supplementary material for: Are exposure to health information and media health literacy associated with fruit and vegetable consumption?
Source: BMC Public Health. 2023 Aug 16;23:1554. doi: 10.1186/s12889-023-16474-1 (PMC10428547; doi:10.1186/s12889-023-16474-1)
Supplement: Supplementary file 1 — Additional file 1. [file 12889_2023_16474_MOESM1_ESM.docx]

[
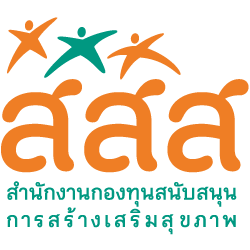
](https://www.google.com/url?sa=i&rct=j&q=&esrc=s&source=images&cd=&ved=2ahUKEwiKhJX76uDjAhXBqY8KHSCfAAEQjRx6BAgBEAU&url=https://www.thaihealth.or.th/&psig=AOvVaw07BldosVxD0W6HRnVL88J9&ust=1564720155099496)[
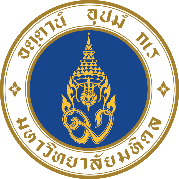
](https://www.google.com/url?sa=i&rct=j&q=&esrc=s&source=images&cd=&ved=2ahUKEwi9xv_i6uDjAhXaknAKHZmTBeMQjRx6BAgBEAU&url=https://mahidol.ac.th/th/logo-corporate-identity/&psig=AOvVaw1e4lIClyj3-tvZ7Xtxj1Sx&ust=1564720106979778)

**The Analysis of Media Landscape, Media Consumption and Media Heath Literacy (MHL) for Thai Children aged 10-14 years Project**

**Questionnaires titled**

**“Media Exposure and Media Helath Literacy among Thai Children Aged 10-14 years”**

This questionnaires were designed and applied from concepts, theroies and literature review both interanational and Thail evidences about media exposure, media health literacy and health behavior of. There are six sections as follows:

Section 1 Media consumption behavior

Section 2 Medid health literacy

Section 3 Health bevahiors

Section 4 Personal information

Section 5 Family data

Section 6 Socio-economic data

**Media health literacy** means abilities to literate health related content about eating and physical activity through media. There are four abilities of media health literacy;

1. **To perceive and understand** means to be able to perceive and recognize that media present heath content and understand the contents, messages or language which media create. (To interpret and understand terminology, symbols and techniques which are used by media.)
2. **To analyze** means to be able to identify components and formats of media contents and messages.
3. **To evaluate** means to be able to evaluate messages which have credibility or benefit or not.
4. **To intent to take action** means to be able to intent to do after exposure to health related content through media and ability to use and apply the health contents from media in order to communicate with others such as parents, peers and teachers.

**Questionnaires titled**

**“Media Exposure and Media Helath Literacy among Thai Children Aged 10-14 years”**

1. Region................................................................................
2. Province.............................................................................
3. District...............................................................................
4. Sub-district........................................................................
5. Address..............................................................................
6. EA…………………………..............................................
7. Housedhold number..........................................................
8. Person ID...........................................................................
9. Interview result

…..Interviewed

…..Refused

…..Not found.

…..Sick / disabled / unable to answer

Data collection on Date............Month...................................Year.................

Name-surname................................................................................................

(Data collector)

**Section 1 Media consumption behavior**

**1. Media consumption**

**1. What media did you ofent expose to in the last week? (Top 3)**

........1. Internet

- 1. facebook
  2. youtube such as Kaekai Slider, Bearhug, Paeng
  3. game such as ROV, Robox, Gareena Free Fire

........2. Television

........3. Raddio

........4. Print media such as comic novels

........5. Outdoor media such as billboards

........6. Specialized media such as brochures, flyers, exhibition

........7. Mobile media (electric train, taxi, train, bus, pickup)

........8. Movie

........9. Other (specify)................................................................

**2. What device do you watch, listen to or read from?**

........Television receiver

........Radio

........Computer

........Laptop/netbook

........Tablet

........Mobile phone (smartphone)

........Other (specify)................................................................

........Print media

........Cartoon book

........Newspaper

........Magazine

........Movie

........Cinema

........DVD/Blu-ray player

**3**. **How often do you expose to these media in the last week?**

........times per day

**4.** **Howlong did you spend to expose to these media in the last week?**

Average time spending.......................hour(s).....................minutes

**2. Source of health information**

**1.** **Have you ever seen health related content about *eating and doing exercis*e or not in the last week?**

........1. No

........2. Yes

**2.** **Are the health content that you expose to all of these? If yes, how much do you like this content?**

|  | **Content** | **Liking** | | |
| --- | --- | --- | --- | --- |
|  |  | **like** | **neutral** | **dislike** |
|  | **Unhealthy content** |  |  |  |
| ........1 | Unhealthy foods such as pizza, fried chicken, fast food, snacks, sweetened beverages (KFC Lay) |  |  |  |
| ........2 | Supplement foods such as food for lost weight, Collagen in diets which makes whitening skin. |  |  |  |
| ........3 | Supplements which strengthen the body, such as foods that help strengthen bones (Anlene) |  |  |  |
| ........4 | Exercise equipment (such as bikes, running shoes of TV Direct) |  |  |  |
|  | **Healthy content** |  |  |  |
| ........1 | Eating healthy food such as eating vegetables and eating all 5 food groups |  |  |  |
| ........2 | Drinking water (such as drinking 8 glasses of water a day), drinking milk (such as drinking milk every day) |  |  |  |
| ........3 | Exercise, such as running (Toon, KaoKonLaKao), Bike for Dad |  |  |  |
| ........4 | Body movements such as dancing (dancing along with K-POP singers) |  |  |  |

**3.** **What channel did you expose to these health content? (Top 3)**

........1. Internet media such as Google, Youtube, social networks (such as Facebook, Line, Instagram)

........2. Television

........3. Mobile phone text messages (SMS)

........4. Personal media such as parents or guardians, relatives, siblings, teachers, friends

........5. Book/ textbook

........6. Other (specify)................................................................

**4. Has anyone persuaded or encourage you to expose to these health related contents?**

........1. No (because it’s my own interest)

........2. Yes (Top 3)

........1. Father/ mother

........2. Grandfather/ grandmother

........3. Uncle/ aunt

........4. Brother/ sister

........5. Friend

........6. Teacher

........7. Public Health officer

........8. Other (specify)................................................................

**5. How often did you expose to these health related contents?**

........times per day

**6. Howlong did you spend to expose to these health related contents last week?**

Average time spending.......................hour(s).....................minutes

**7. What time do you expose to these health related contents? (Order 1-2)**

........After midnight (00.01-04.00 hrs.)

........Morning (04.01-10.00 hrs.)

........Lunch (13.01-16.00 hrs.)

........Afternoon (13.01-16.00 hrs.)

........Evening (16.01-20.00 hrs.)

........Night (20.01-24.00 hrs.)

**8. Has anyone ever told you that food which you ate have benefit for your health and growth?**

........1. No (Skip to Q9)

........2. Yes (Top 3)

........1. Father/ mother

........2. Grandfather/ grandmother

........3. Uncle/ aunt

........4. Brother/ sister

........5. Friend

........6. Teacher

........7. Public Health officer

........8. Other (specify)................................................................

**9. Has anyone ever told you about weight controlling information, or controlling the amount of food and beverages consumed inorder to be short or obese?**

........1. No (Skip to Q10)

........2. Yes (Top 3)

........1. Father/ mother

........2. Grandfather/ grandmother

........3. Uncle/ aunt

........4. Brother/ sister

........5. Friend

........6. Teacher

........7. Public Health officer

........8. Other (specify)................................................................

**10. Has anyone ever told you about doing exercise information or body movement in order to strengthen your body?**

........1. No (Skip to Q11)

........2. Yes (Top 3)

........1. Father/ mother

........2. Grandfather/ grandmother

........3. Uncle/ aunt

........4. Brother/ sister

........5. Friend

........6. Teacher

........7. Public Health officer

........8. Other (specify)................................................................

**11. Has anyone ever told you that** **playing online games for a long time ทay cause weight gain or obesity**?

........1. No (Skip to Q12)

........2 .Yes (Top 3)

........1. Father/ mother

........2. Grandfather/ grandmother

........3. Uncle/ aunt

........4. Brother/ sister

........5. Friend

........6. Teacher

........7. Public Health officer

........8. Other (specify)................................................................

**12.** **If you want to find information about the *5* main food groups, where are the** **top 3 places to** **find this information?**

........1. Internet media such as Google, Yoytube, social networks (such as Facebook, Line, Instagram)

........2. Television

........3. Mobile phone text messages (SMS)

........4. Personal media such as parents or guardians, relatives, siblings, teachers, friends

........5. Book/ textbook

........6. Other (specify)................................................................

**13. If you want to find information about exercise or body movement, where do I** **find the most top 3** **such information from?**

........1. Internet media such as Google, Yoytube, social networks (such as Facebook, Line, Instagram)

........2. Television

........3. Mobile phone text messages (SMS)

........4. Personal media such as parents or guardians, relatives, siblings, teachers, friends

........5. Book/ textbook

........6. Other (specify)................................................................

**Section 2 Media health literacy (4 clips)**

Research team selected media which showed eating and doing exercise content from focus group discusiion among children aged 10-14 years.

**Evaluation:** Participants see four video clips and then, they answer the questions in order to access media health literacy skills. There are four skills of media health literacy as follows:

1. **To perceive and understand** means to be able to perceive and recognize that media present heath content and understand the contents, messages or language which media create. (To interpret and understand terminology, symbols and techniques which are used by media.)
2. **To analyze** means to be able to identify components and formats of media contents and messages.
3. **To evaluate** means to be able to evaluate messages which have credibility or benefit or not.
4. **To intent to take action** means to be able to intent to do after exposure to health related content through media and ability to use and apply the health contents from media in order to communicate with others such as parents, peers and teachers.

**Video clip No.1: Eating 2:1:1 (31 seconds)**

| **Piture** | **Sound** |
| --- | --- |
| 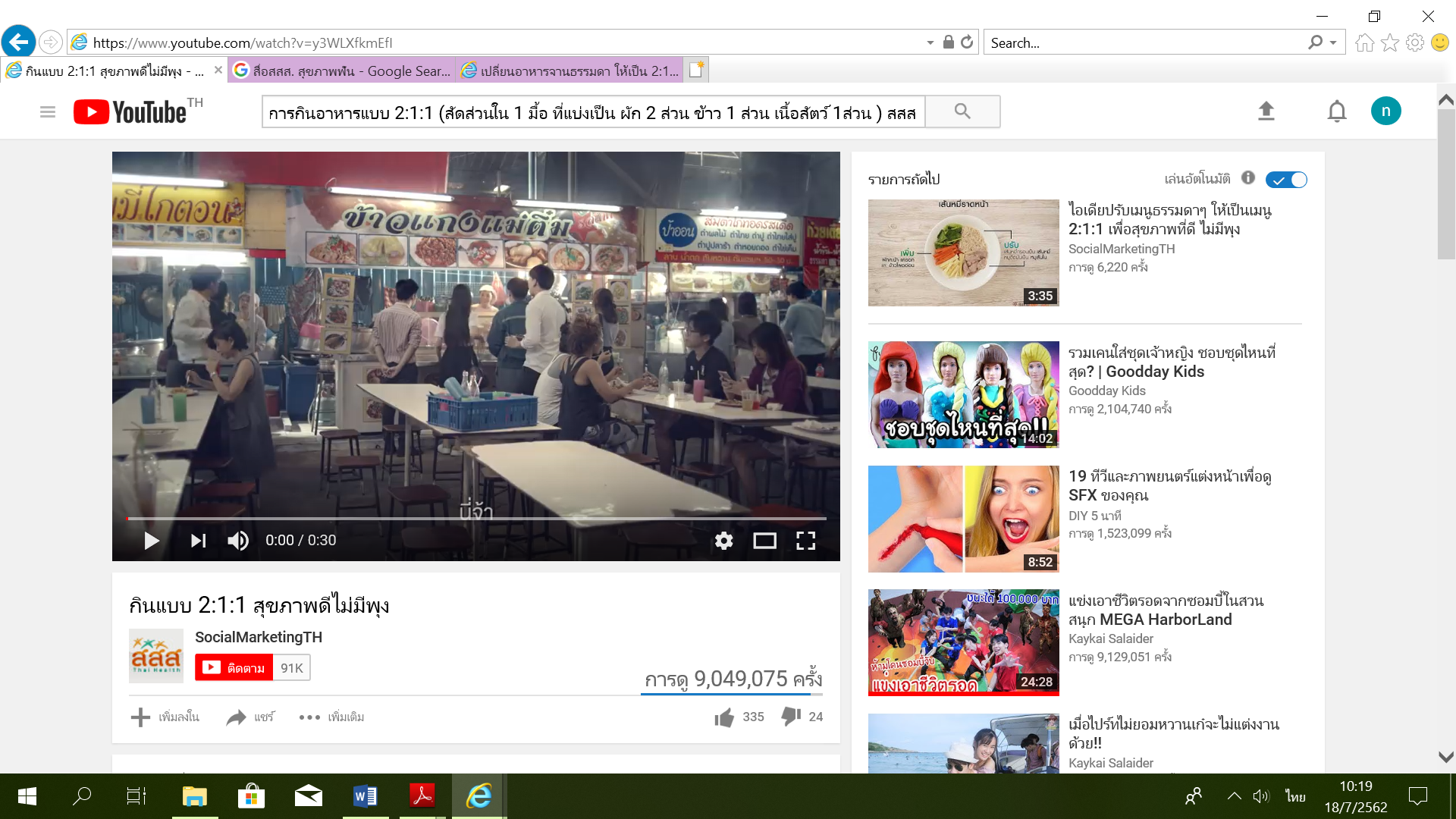 | Woman: here……what do you want to eat? |
| 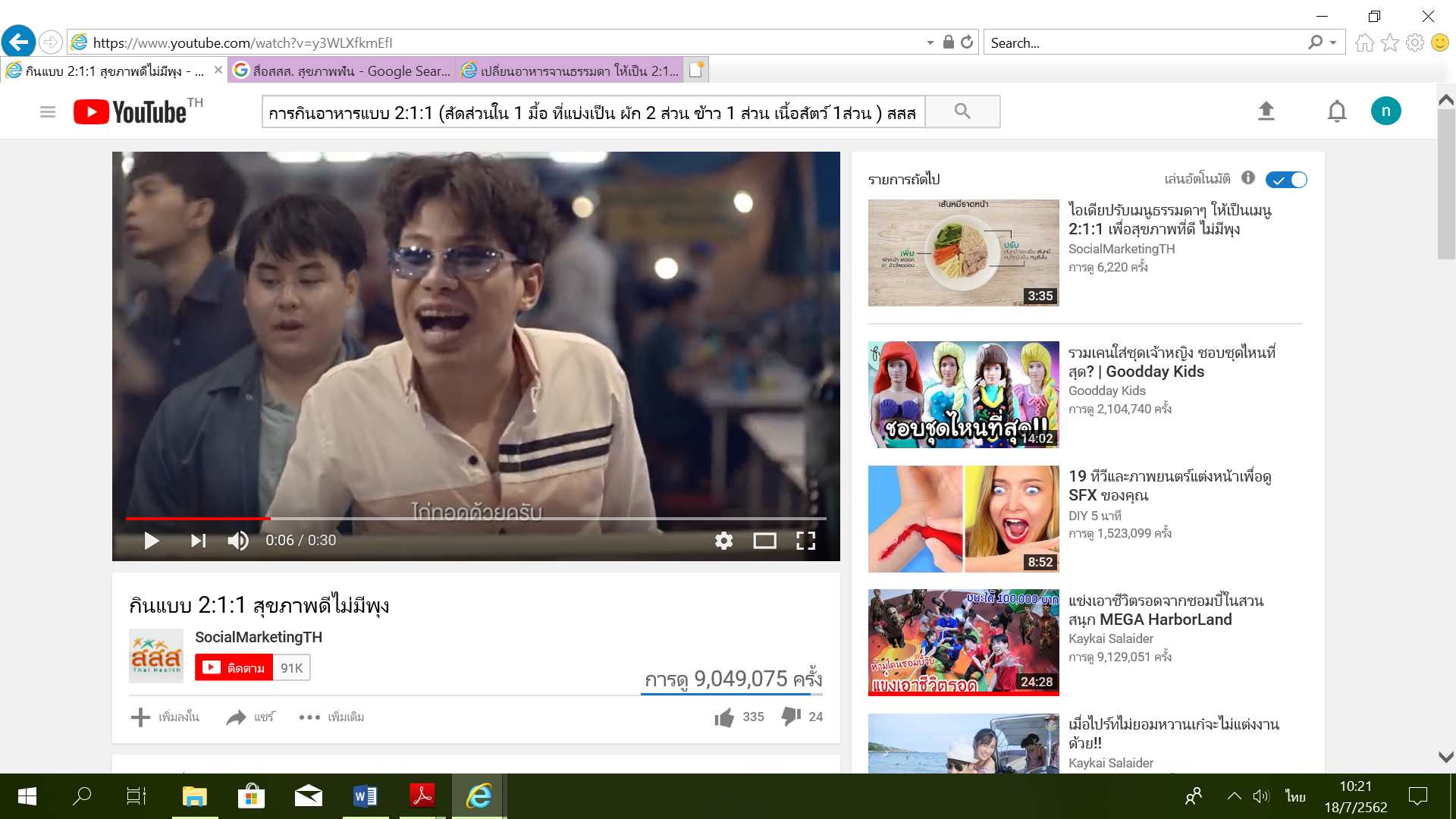 | Man1: fried chicken  Man2: Sweet pork and fired chicken as well. |
| 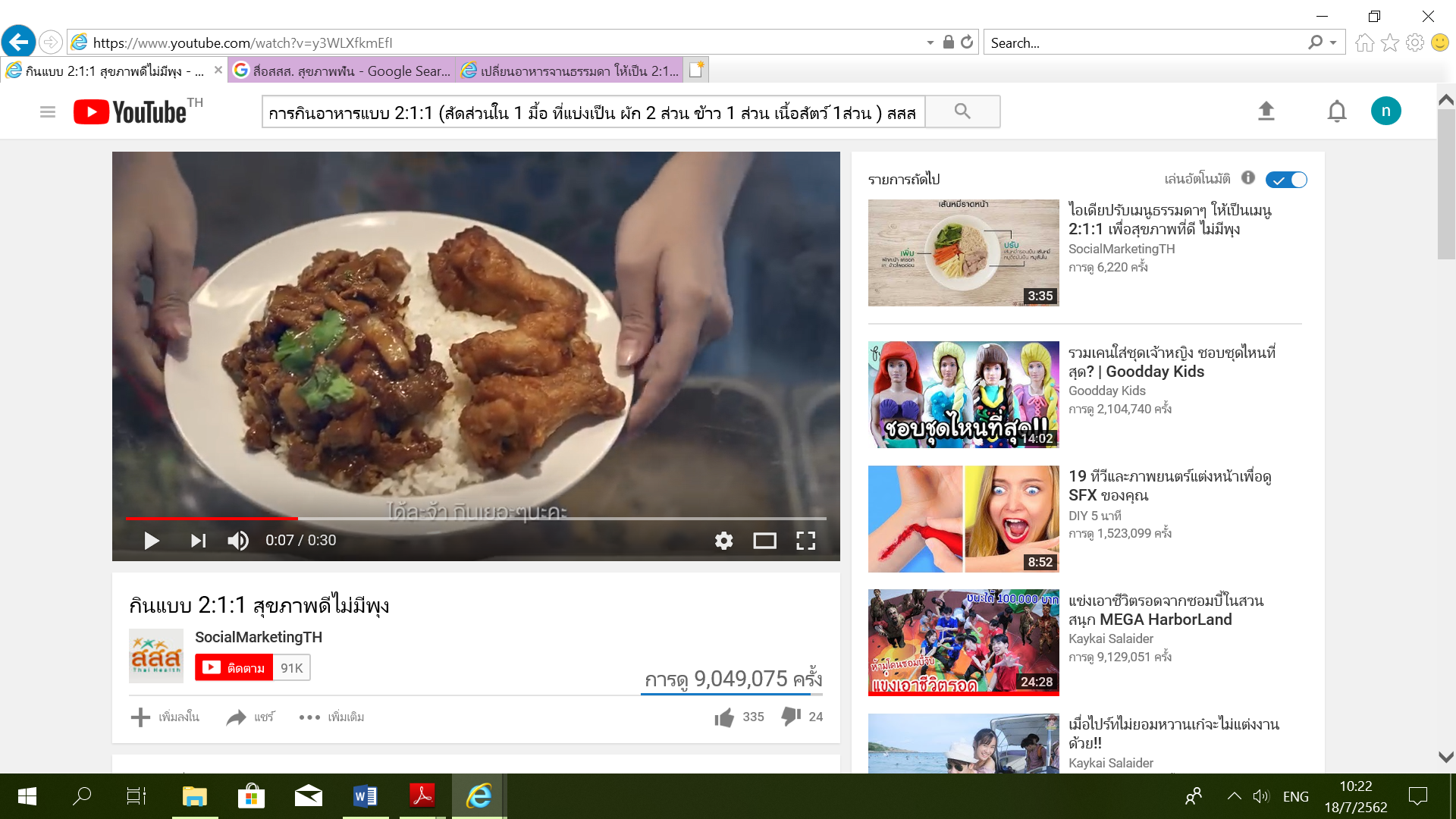 | Woman: Here you are and enjoy to eat. |
| 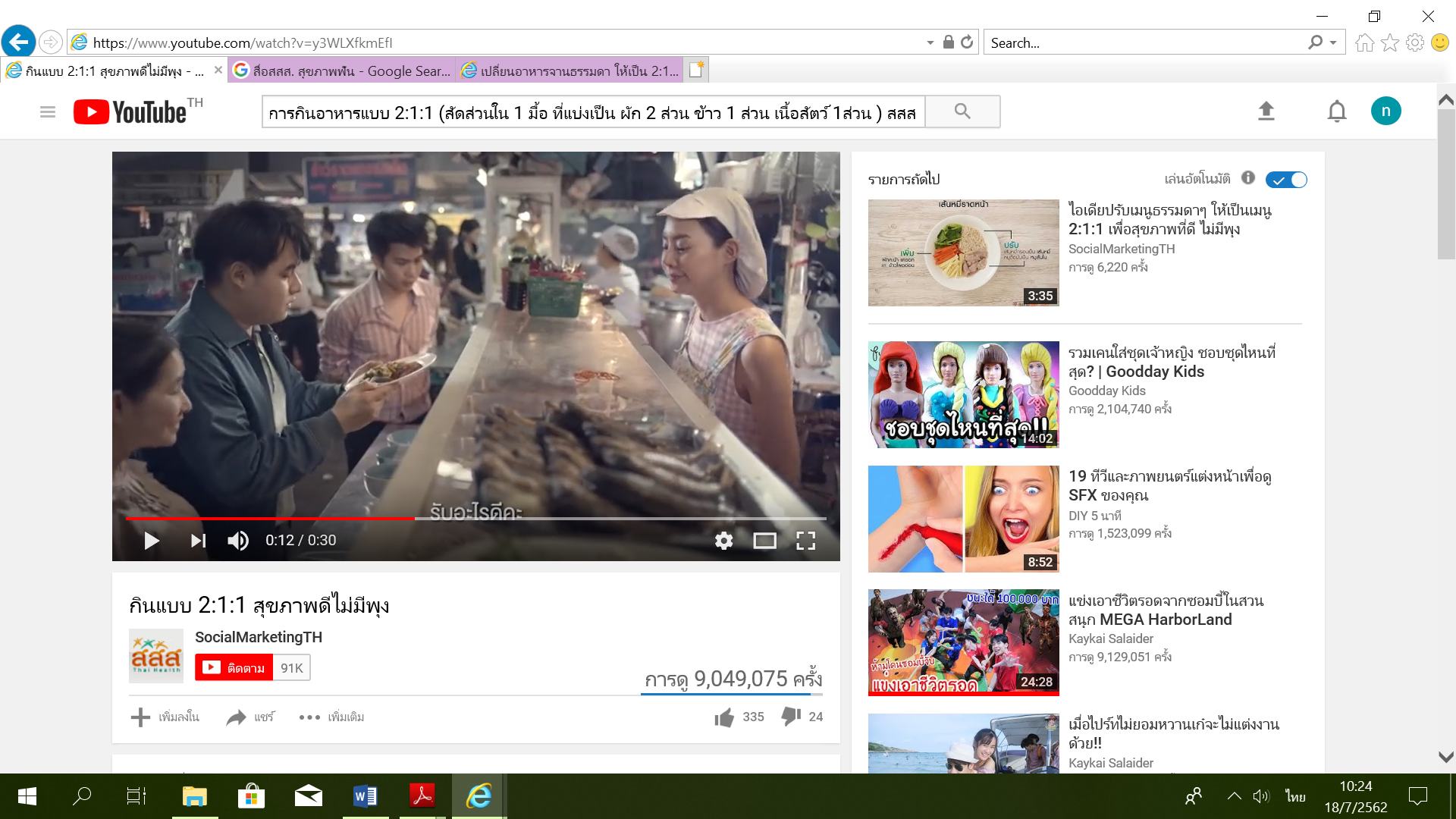 | Man1: Um…m This is not my order. |
| 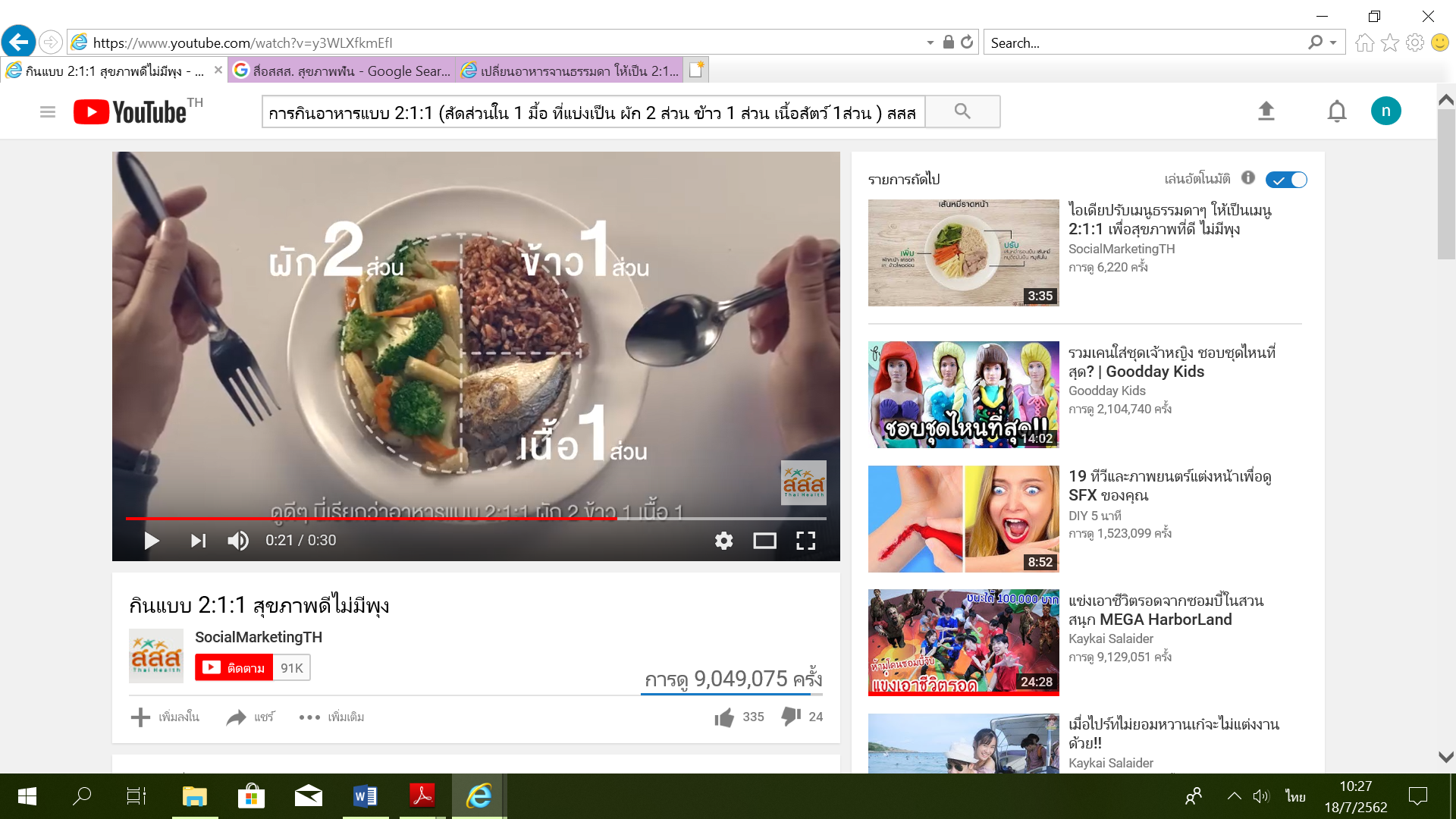 | Description: This is call eat 2:1:1  veggie 2 rice 1 meat 1  If she doesn’t love him, she won’t give this dish to him. |
| 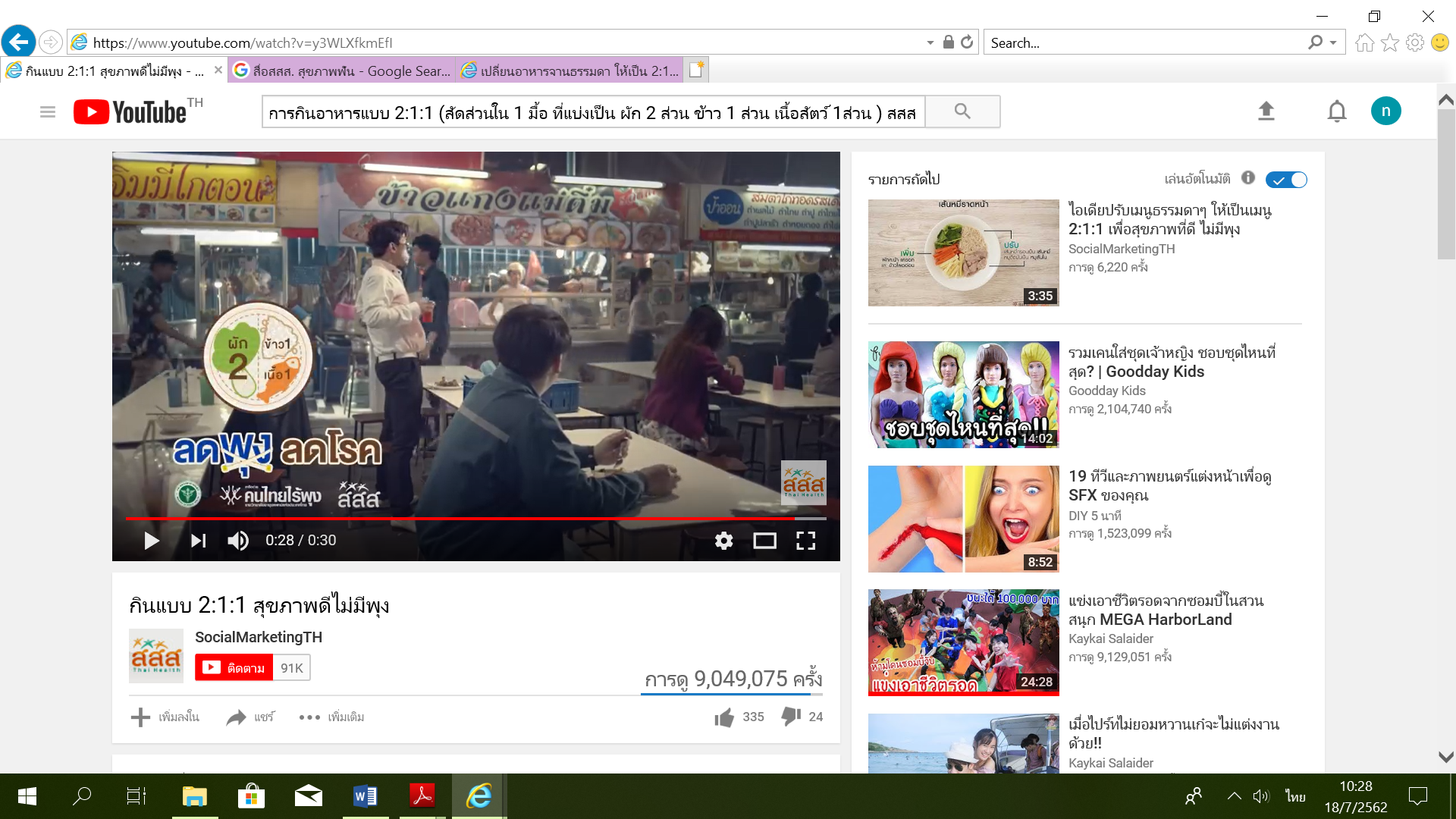 | Description: eat 2:1:1 and do exercise. Then you are not pot-bellies. |

**1. Do you know this clip?**

........1. No

........2. Yes

**2. Please answer these questions**

| **MHL** | **Question** | **Answer** | | | |
| --- | --- | --- | --- | --- | --- |
| **Perceive and understand** | 1. What product does this advertising sell? | Rice and curry | Fried chicken | Do not sell any products | A la carte |
|  | 1. What content has appeared in the media that you just watched, to present a story about? | Line up in line to buy food | Eat 2: 1: 1 | Food stalls | The love between the seller and the customer |
|  | 1. Is there the content about health appear in the media? | Yes | No |  |  |
| **Analyze** | 1. What would be the purpose of this advertisement? | To queue up to buy food | To suggest eating that does not cause obesity | To promote healthy eating | To eat a lot of vegetables. |
|  | 1. Who are created this video clip? | Advertising agency | The company or product owner | Government agency | Both government agency and advertising agency |
|  | 1. Where does the content and information from the presenter or the presenter in the advertisement come from? | True heart of the presenter ง | Dialogue written by the advertiser | Facts reflected by experts | Both dialogue written by the advertiser and facts reflected by experts |
|  | 1. Who is target audience? | Children | Teenagerss | Adults | Everyone |
|  | 1. Who are get benefit from this video clip? | Food restaurant | Audiences | Government agency | Advertising agency |
| **Evaluate** | 1. Do you like this video clip? | Yes | No |  |  |
|  | 1. Is this video clip reliable? | Yes | No |  |  |
|  | 1. Is this video clip benefit for you? | Yes | No |  |  |
| **Intent to take act** | 1. After watching this video clip, do you intend to eat 2 parts of vegetables, 1 part of rice, 1 part of meat or not? | Yes | No |  |  |
|  | 1. After watching this video clip, do you intend to tell your parents or guardians to eat 2 parts of vegetables, 1 part of rice, 1 part of meat or not? | Yes | No |  |  |
|  | 1. After watching this video clip, do you intend to tell your friends to eat 2 parts of vegetables, 1 part of rice, 1 part of meat or not? | Yes | No |  |  |
|  | 1. Will you take the information from this video clip to do in daily life or not, such as education, reporting or homework to send teachers? | Yes | No |  |  |

# Video clip No.2: Begin again (1 minute)

| **Picture** | **Sound** |
| --- | --- |
| 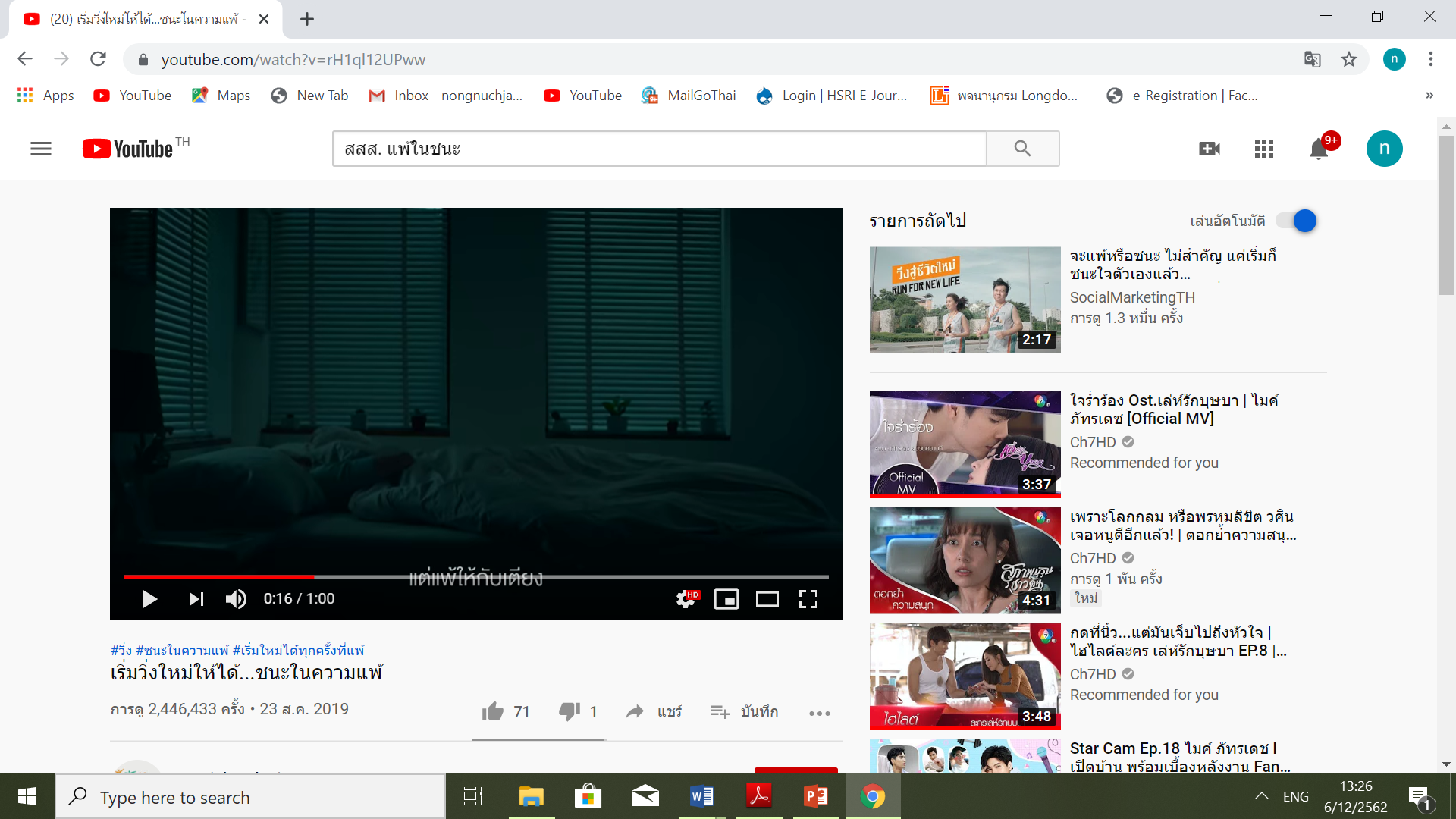 | Woman: I call myself that loser. I would like to run but I still sleep on my bed. |
| 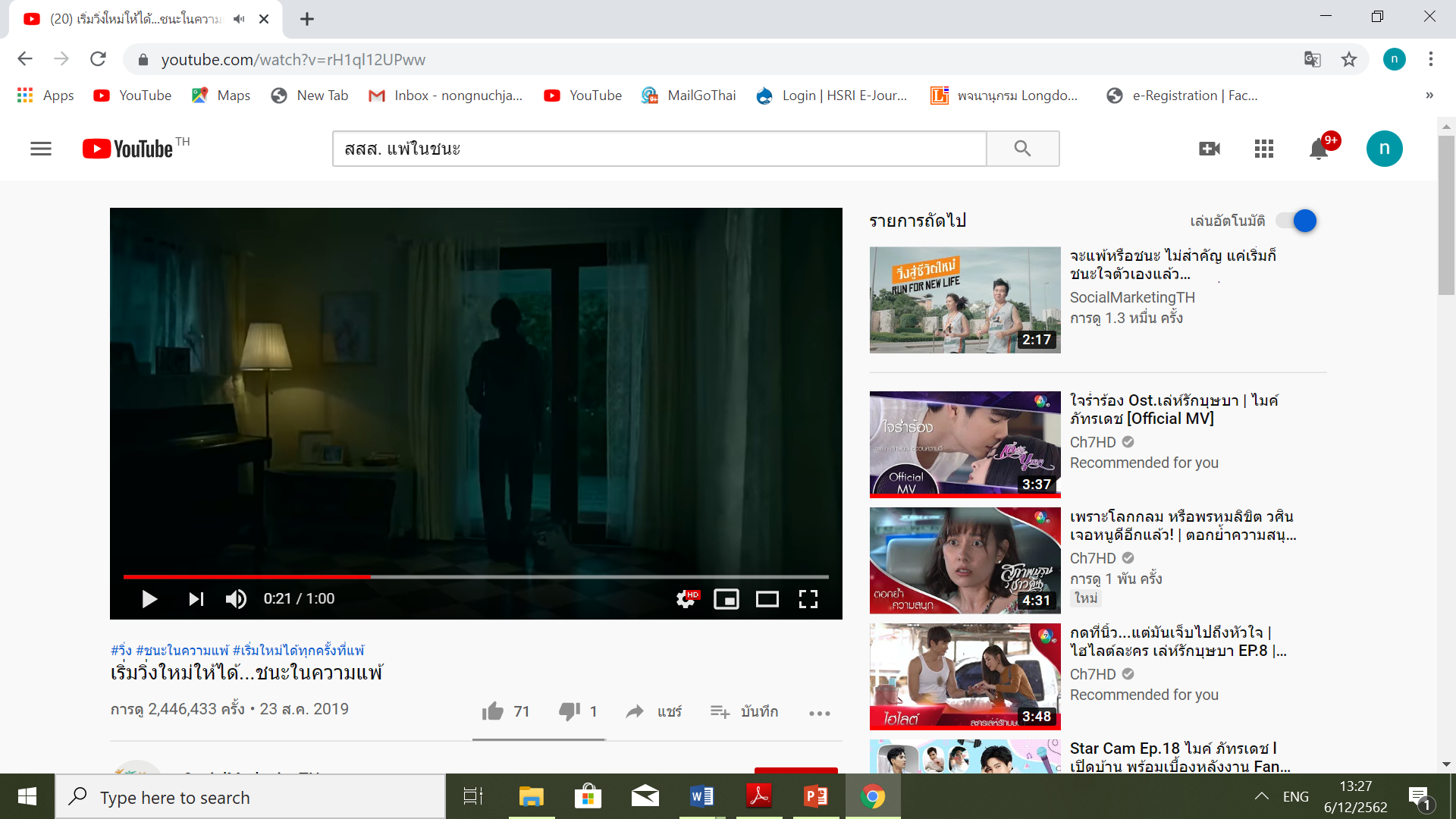 | Woman: I cannot run because of raininhg. So, I’m a loser. |
| 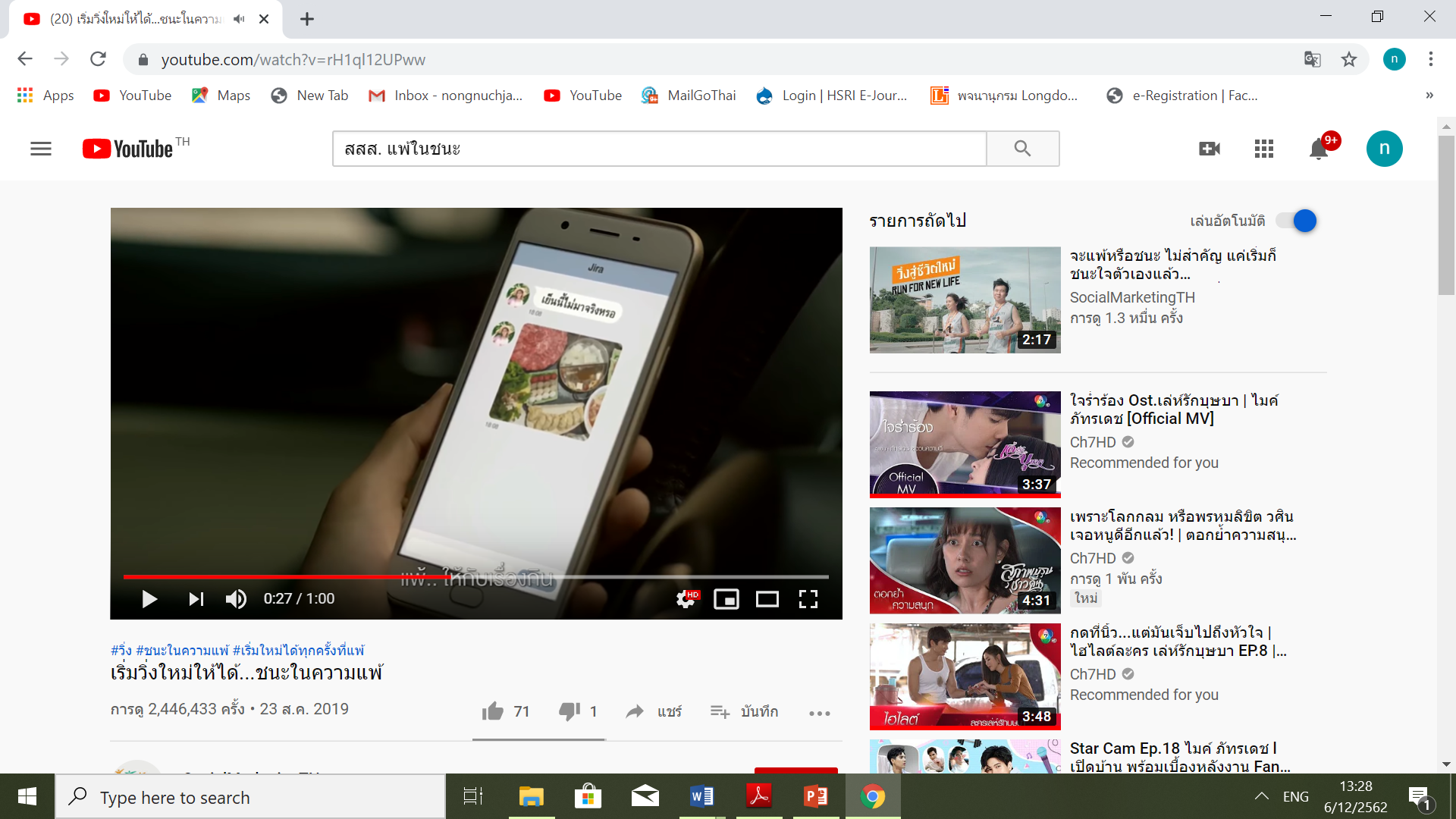 | Woman: I cannot run because I would like to eat food. So, I’m a loser. |
| 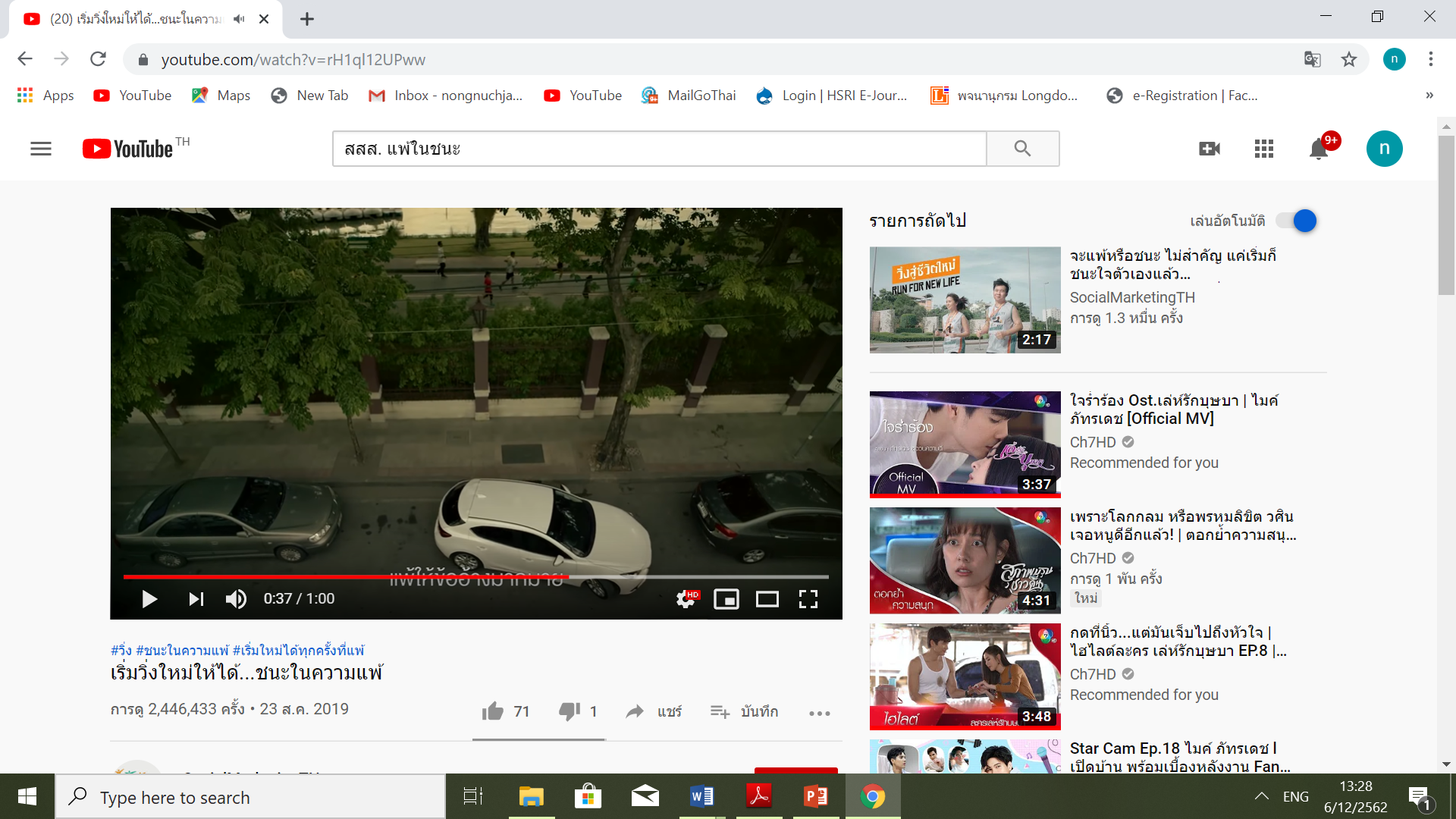 | Music  Woman: I have many excuses to stop running. So, I’m a loser. |
| 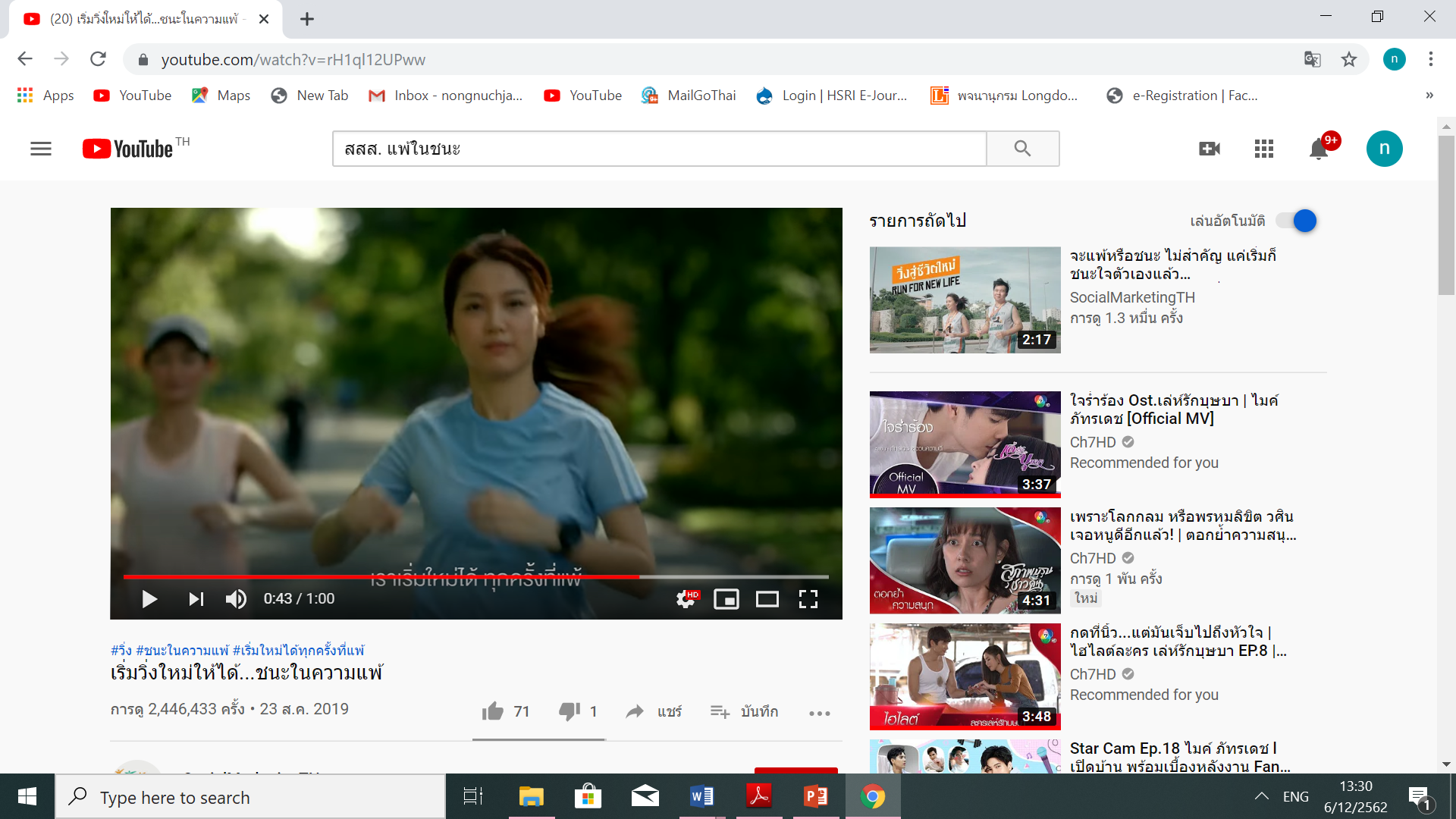 | Music  Woman: But I can strat running again, eventhough I did run in the past.  Woman2: Today, you run many rounds. |
| 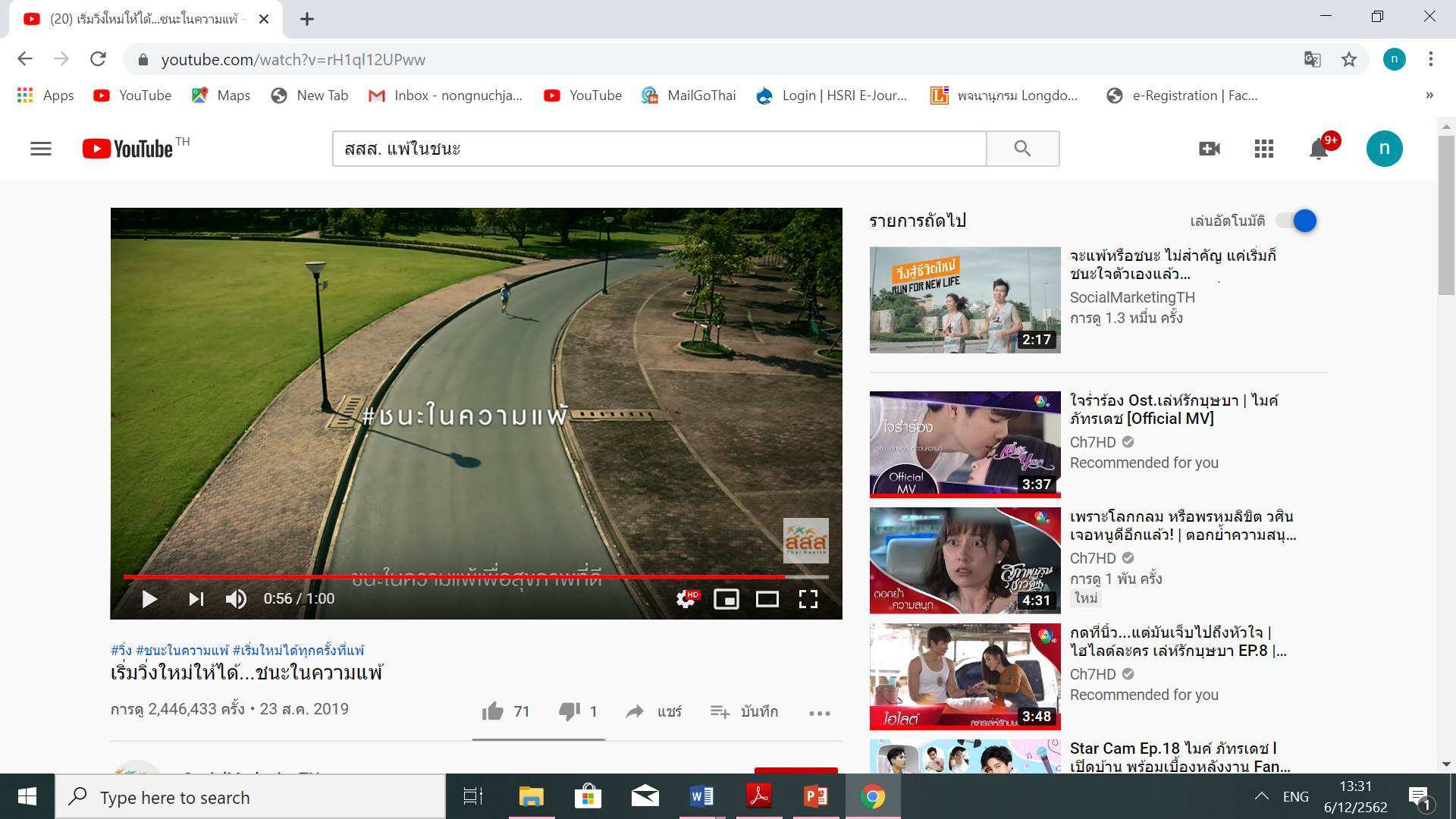 | Description: Win in the lostfor healthy  Thai Health Promotion Foundation |

**1. Do you know this clip?**

........1. No

........2. Yes

**2. Please answer these questions**

| **MHL** | **Question** | **Answer** | | | |
| --- | --- | --- | --- | --- | --- |
| **Perceive and understand** | 1. What product does this advertising sell? | Running shoes | Mobile phone | Ordering food via mobile phone | Do not sell any products |
|  | 1. What content has appeared in the media that you just watched, to present a story about? | Raining and traffic congestion | Sleep lover | Food lover | Exercise intention |
|  | 1. Is there the content about health appear in the media? | Yes | No |  |  |
| **Analyze** | 1. What would be the purpose of this advertisement? | To stay home when it’s raining | เTo sleep | To prevent obesity | To encourage to do exercise |
|  | 1. Who are created this video clip? | Advertising agency | Mobile phone company | Government agency | Both government agency and advertising agency |
|  | 1. Where does the content and information from the presenter or the presenter in the advertisement come from? | True heart of the presenter ง | Dialogue written by the advertiser | Facts reflected by experts | Both dialogue written by the advertiser and facts reflected by experts |
|  | 1. Who is target audience? | Children | Teenagers | Adults | Everyone |
|  | 1. Who are get benefit from this video clip? | Mobile phone company | Shoe company | Government agency | Audiences |
| **Evaluate** | 1. Do you like this video clip? | Yes | No |  |  |
|  | 1. Is this video clip reliable? | Yes | No |  |  |
|  | 1. Is this video clip benefit for you? | Yes | No |  |  |
| **Intent to take act** | 1. After watching this video clip, do you intend to run or not? | Yes | No |  |  |
|  | 1. After watching this video clip, do you intend to tell your parents or guardians to run or not? | Yes | No |  |  |
|  | 1. After watching this video clip, do you intend to tell your friends to run or not? | Yes | No |  |  |
|  | 1. will you take the information from this video clip to do in daily life or not, such as education, reporting or homework to send teachers? | Yes | No |  |  |

**Video clip No.3: Snack (16 seconds)**

| **Picture** | **Sound** |
| --- | --- |
| 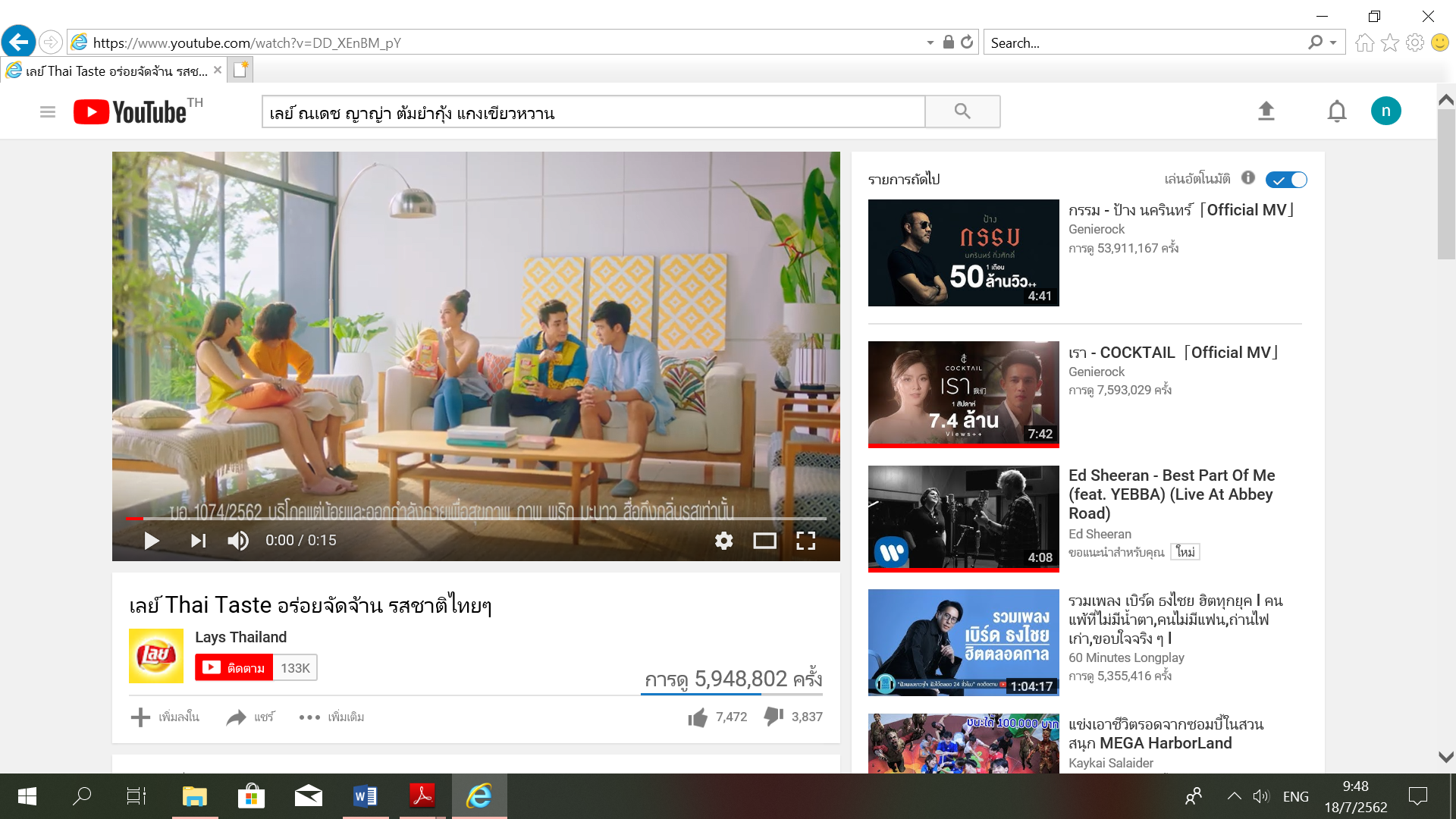 | Man: What is spicy? |
| 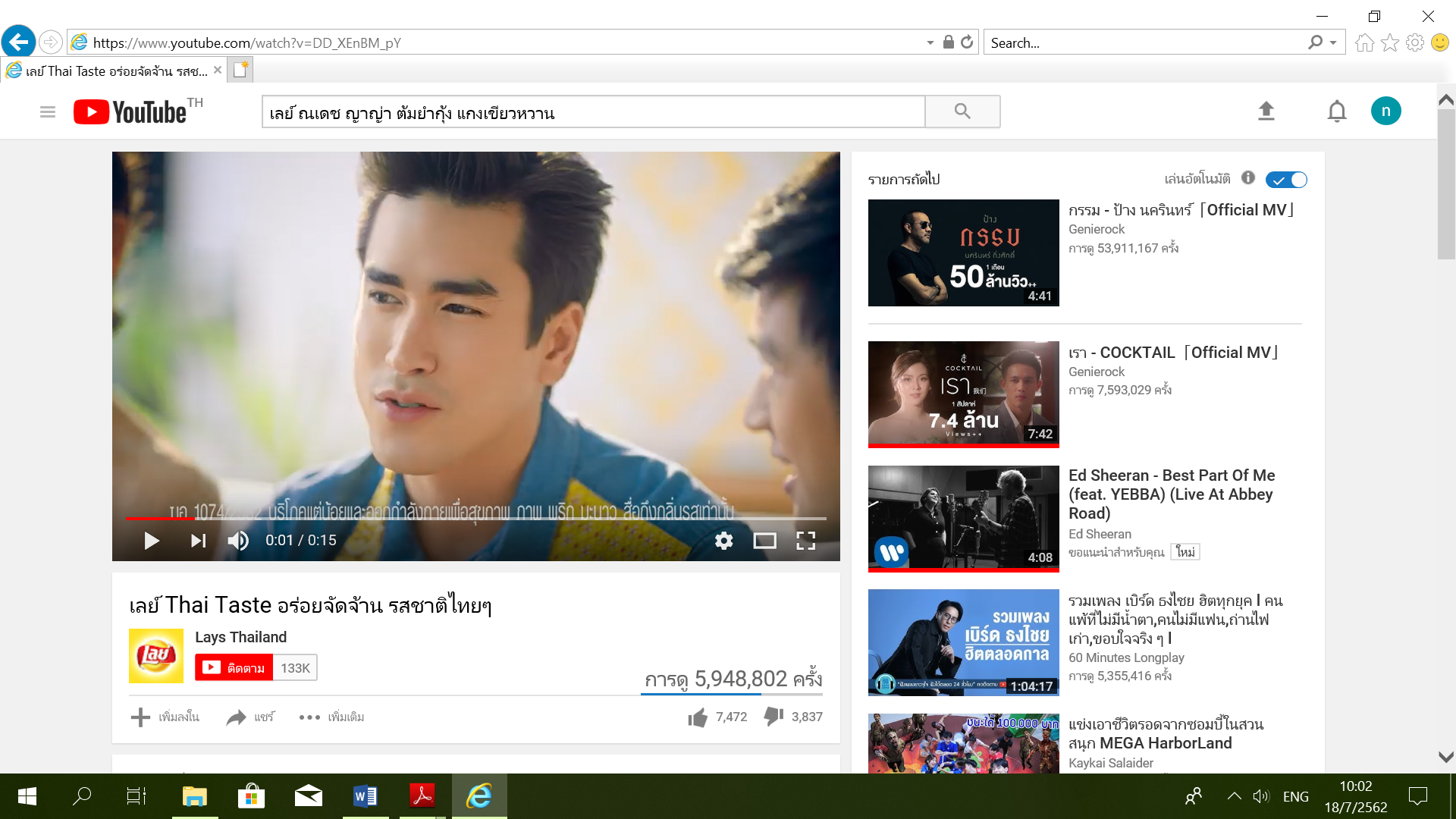 | Actor: Spicy and sour |
| 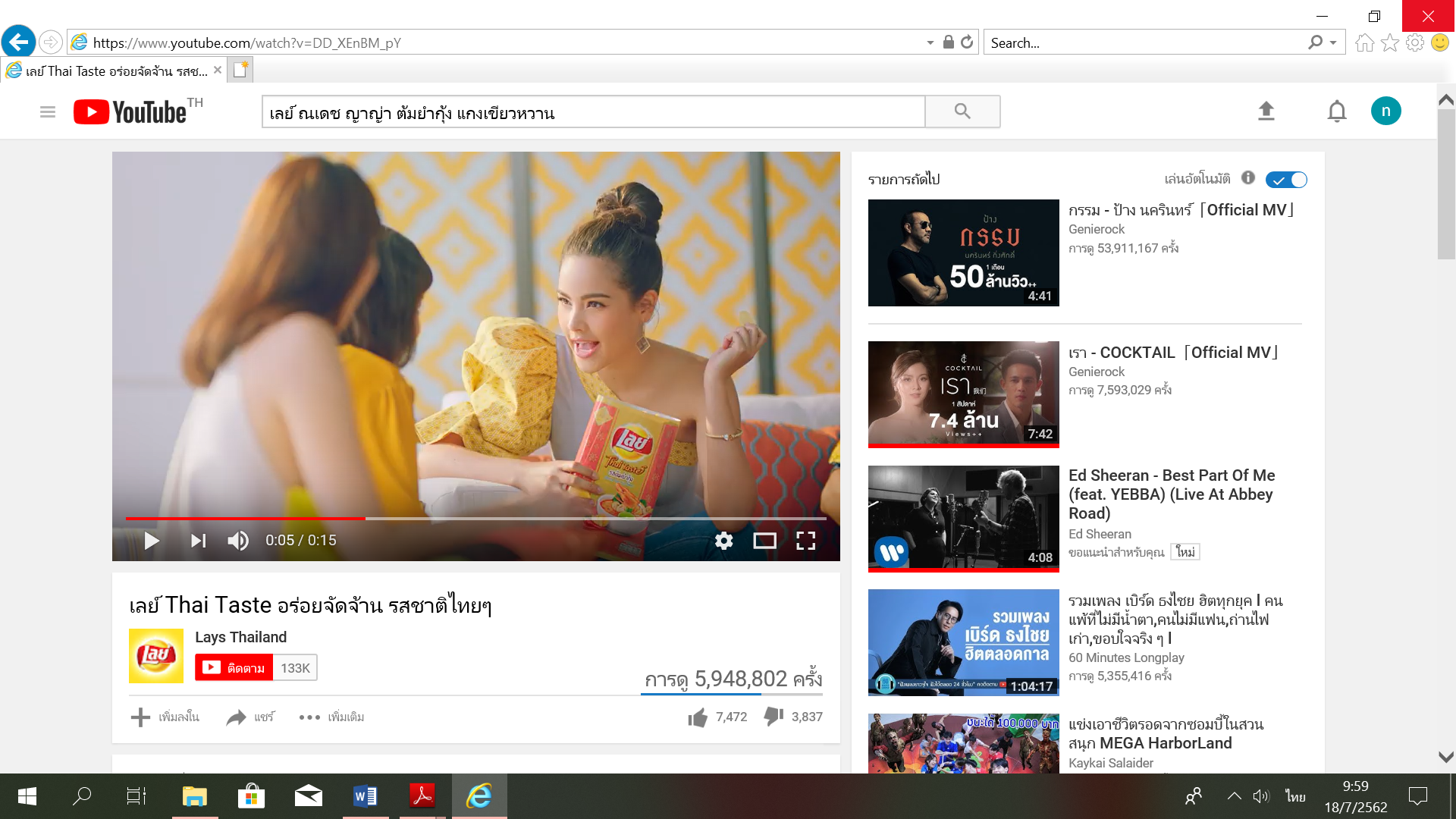 | Actress: full of flavor |
| 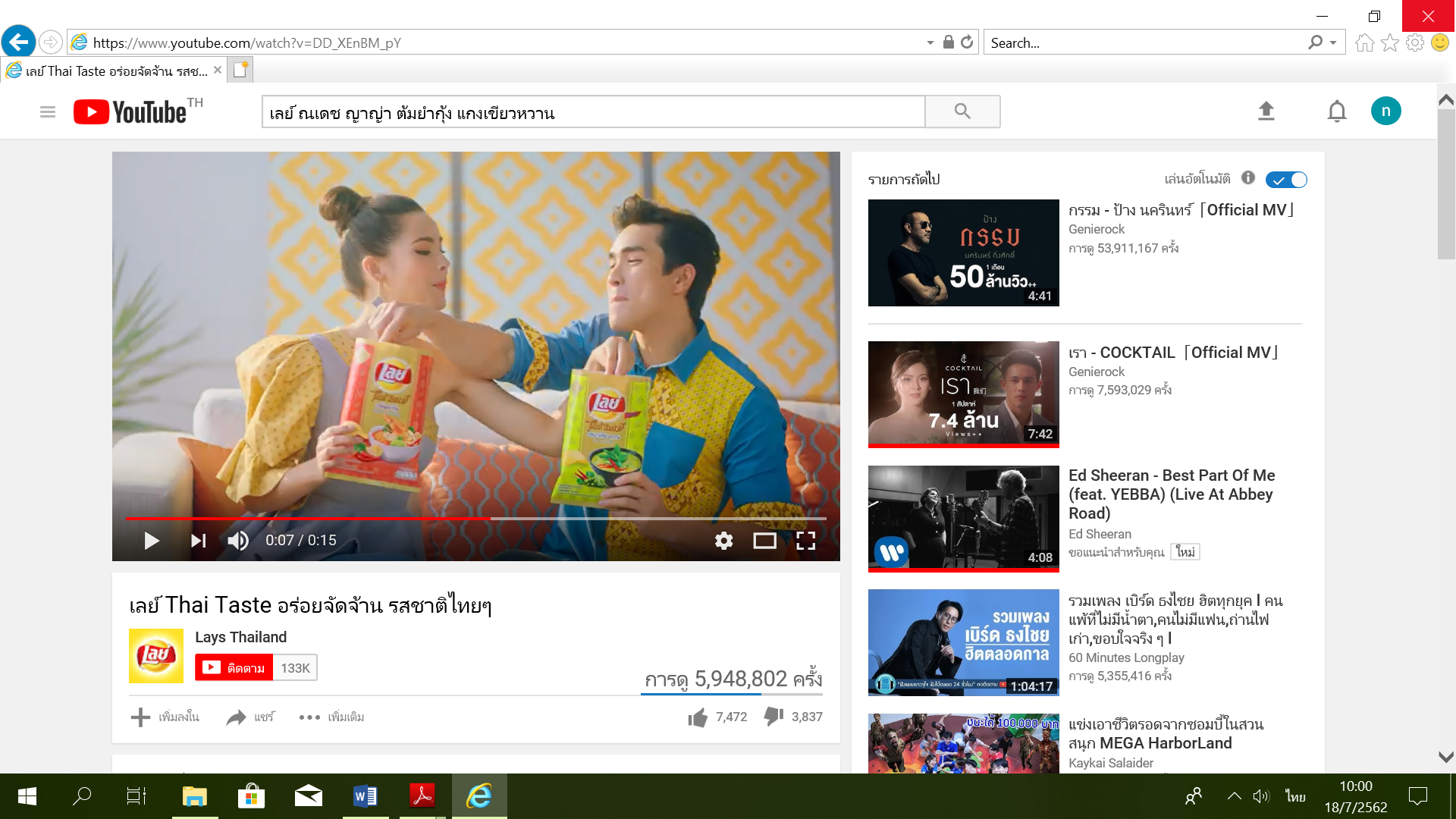 | Actor and actress say: Yummy |
| 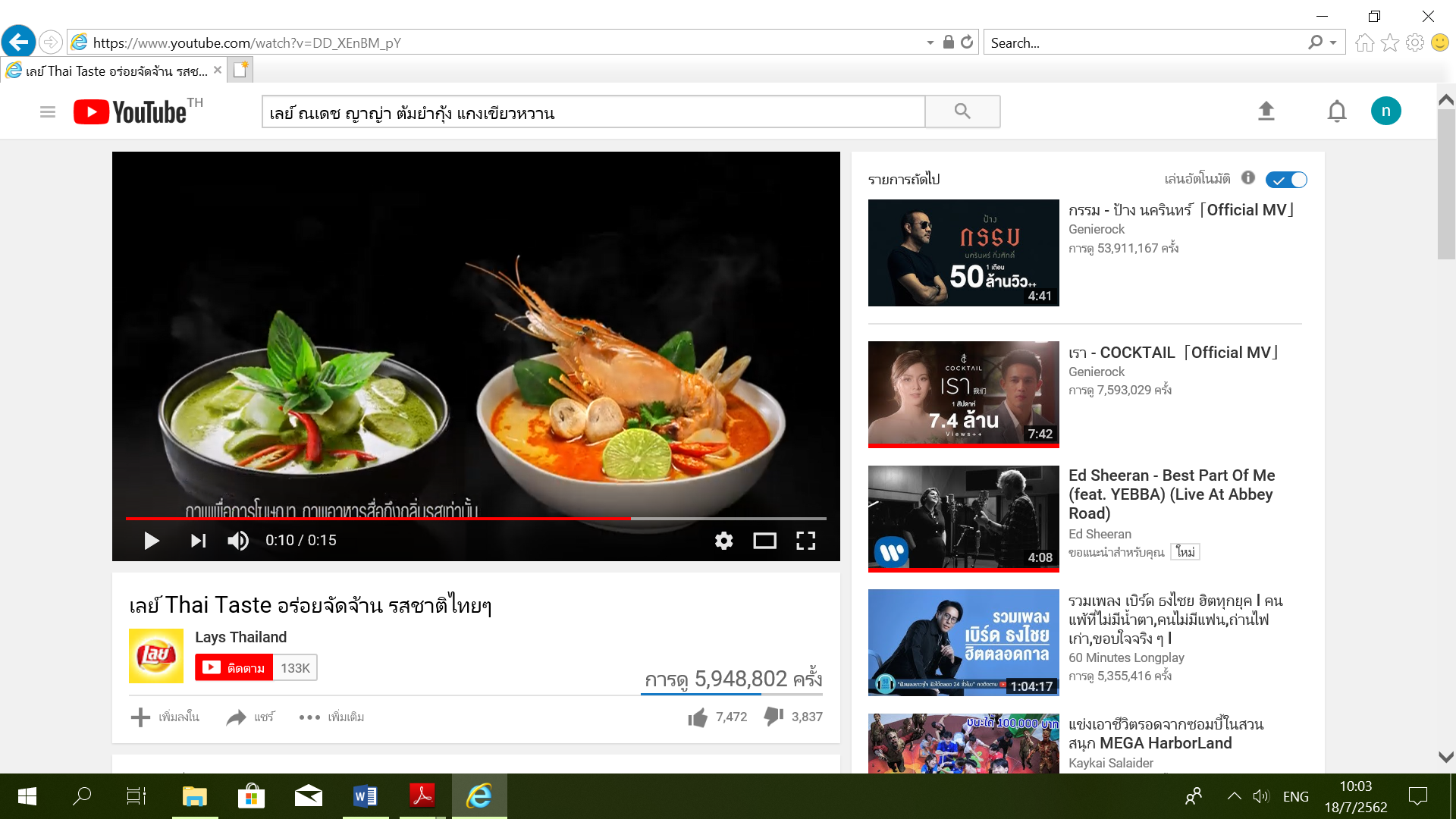 | Description: New! Lay Thai taste: Tom Yum Kung and Green curry |
| 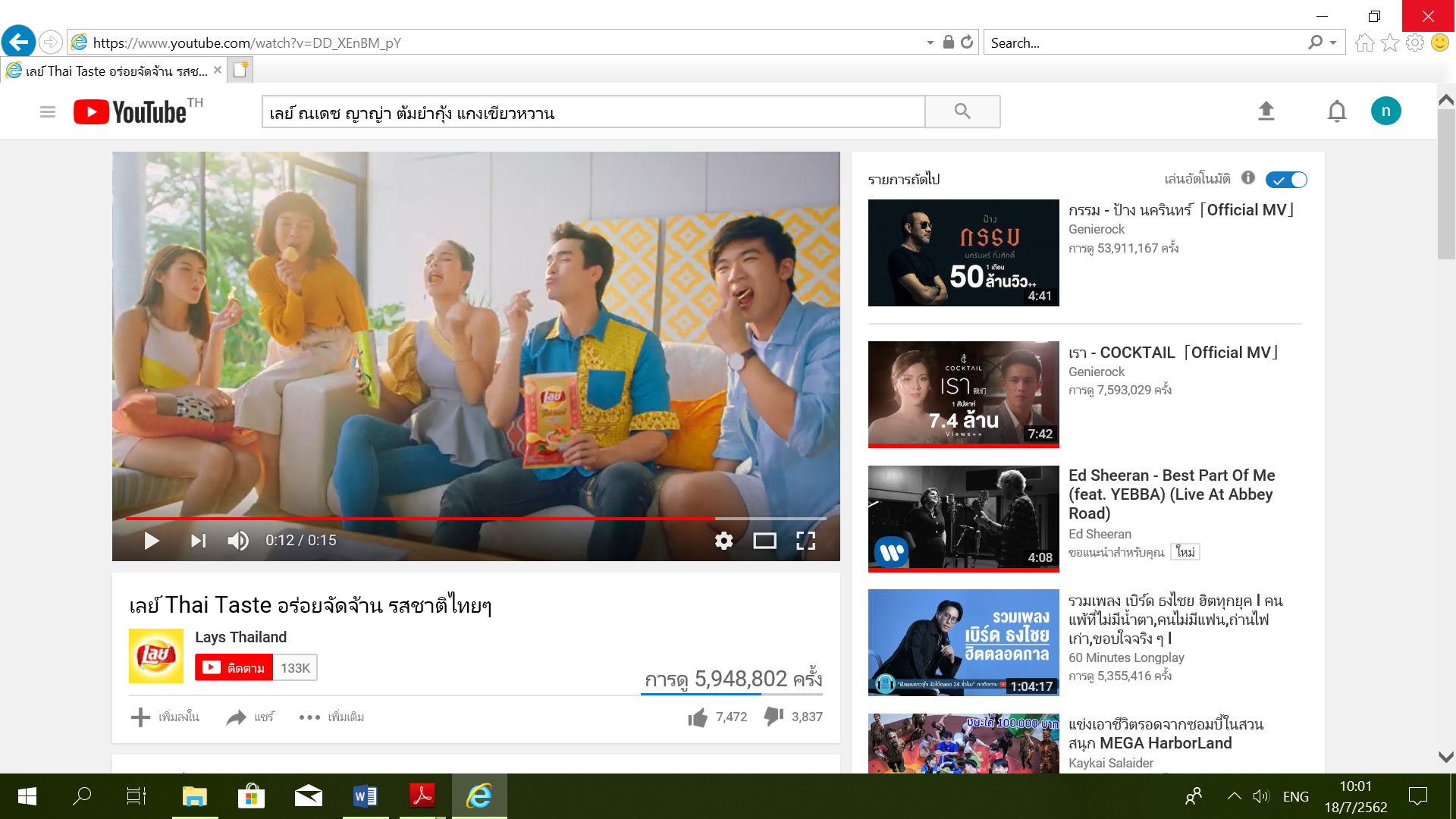 | Everyone: Thai taste, Everyone fall in love it, Try it. |
| 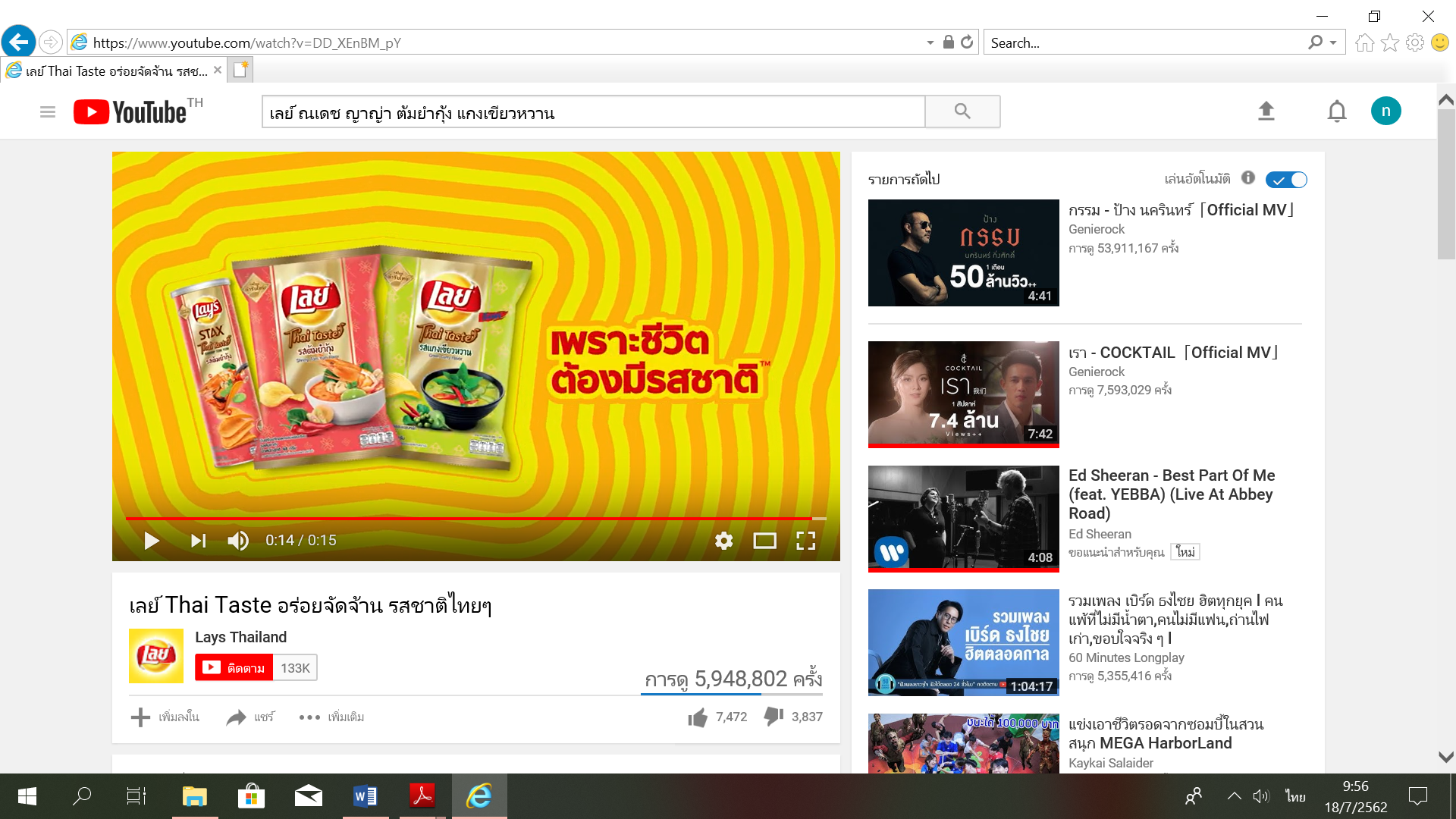 | Music: Life must taste. |

**1. Do you know this clip?**

........1. No

........2. Yes

**2. Please answer these questions**

| **MHL** | **Question** | **Answer** | | | |
| --- | --- | --- | --- | --- | --- |
| **Perceive and understand** | 1. What product does this advertising sell? | Thai food | Spicy potato chips | New Thai food taste of potato chips | Do not sell any products |
|  | 1. What content has appeared in the media that you just watched, to present a story about? | Food | Party | Eating potato chips with friends | New Thai food taste of potato chips |
|  | 1. Is there the content about health appear in the media? | Yes | No |  |  |
| **Analyze** | 1. What would be the purpose of this advertisement? | To recommend product | to show eating | To show deliciousness | to persuade audience to buy the product |
|  | 1. Who are created this video clip? | Advertising agency | The company or product owner | Government agency | Both the company or product owner and advertising agency |
|  | 1. Where does the content and information from the presenter or the presenter in the advertisement come from? | True heart of the presenter ง | Dialogue written by the advertiser | Facts reflected by experts | all are coorect |
|  | 1. Who is target audience? | Children | Teenagers | Adults | Elderly people |
|  | 1. Who are get benefit from this video clip? | Advertising agency | The company or product owner | Presenters | Audiences |
| **Evaluate** | 1. Do you like this video clip? | Yes | No |  |  |
|  | 1. Is this video clip reliable? | Yes | No |  |  |
|  | 1. Is this video clip benefit for you? | Yes | No |  |  |
| **Intent to take act** | 1. After watching this video clip, do you intend to buy or not? | Yes | No |  |  |
|  | 1. After watching this video clip, do you intend to tell your parents or guardians to buy or not? | Yes | No |  |  |
|  | 1. After watching this video clip, do you intend to tell my friends to buy or not? | Yes | No |  |  |
|  | 1. Will you take the information from this video clip to doin daily life or not, such as education, reporting or homework to send teachers? | Yes | No |  |  |

**Video clip No.4: Fried chicken (15 seconds)**

| **Picture** | **Sound** |
| --- | --- |
| 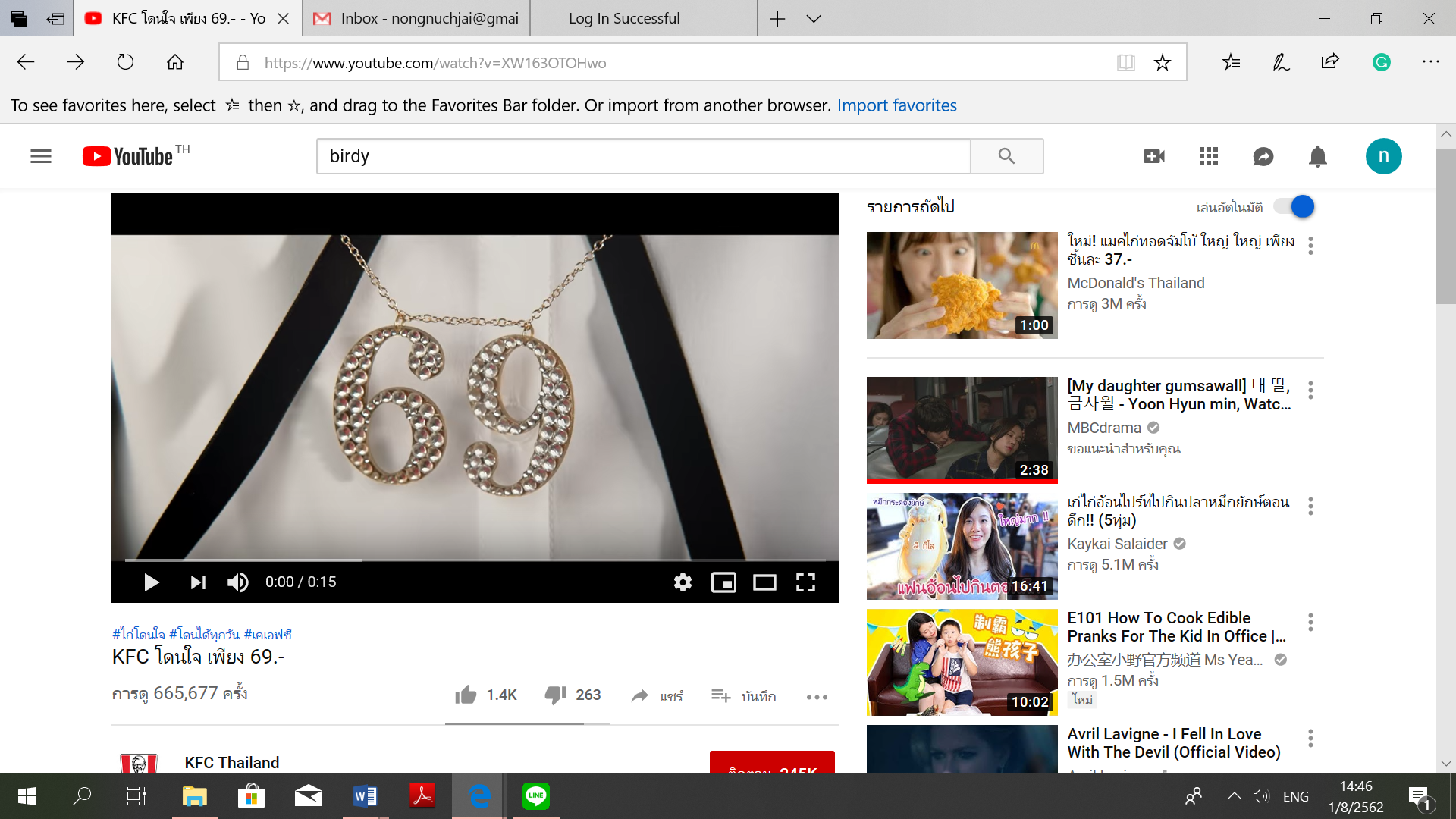 | Music |
| 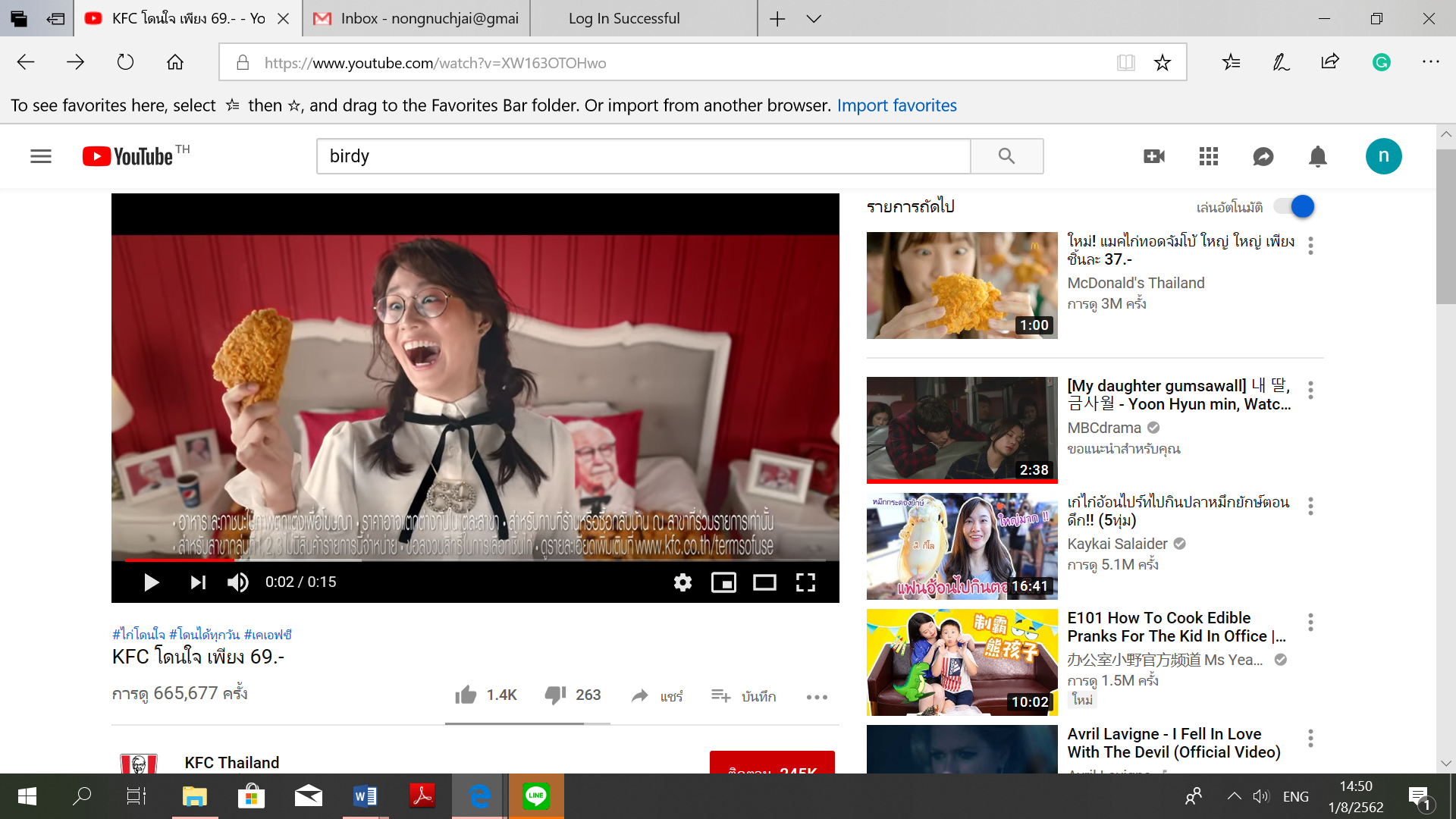 | Music |
| 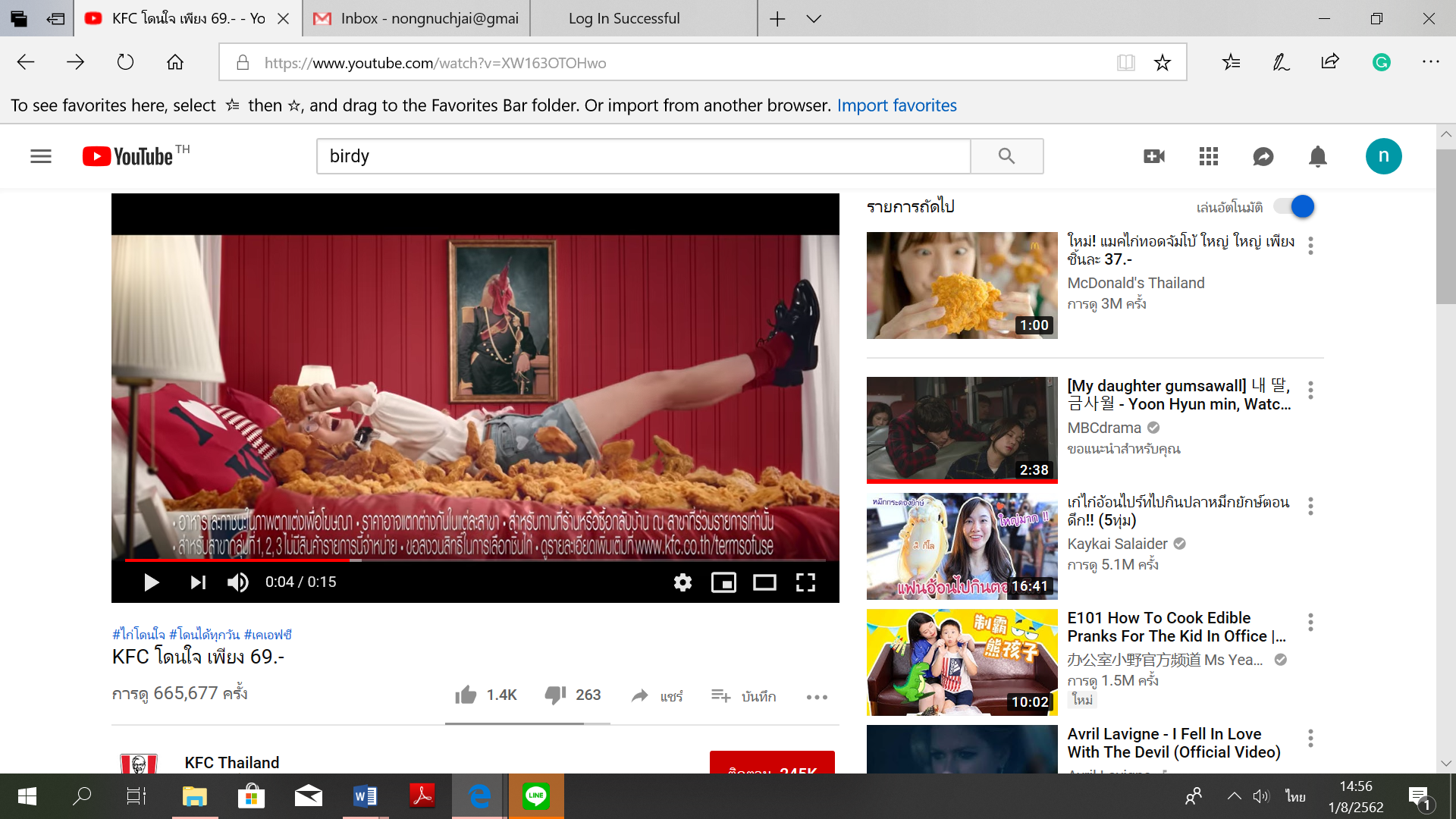 | Description:: Lay down your favorite food. |
| 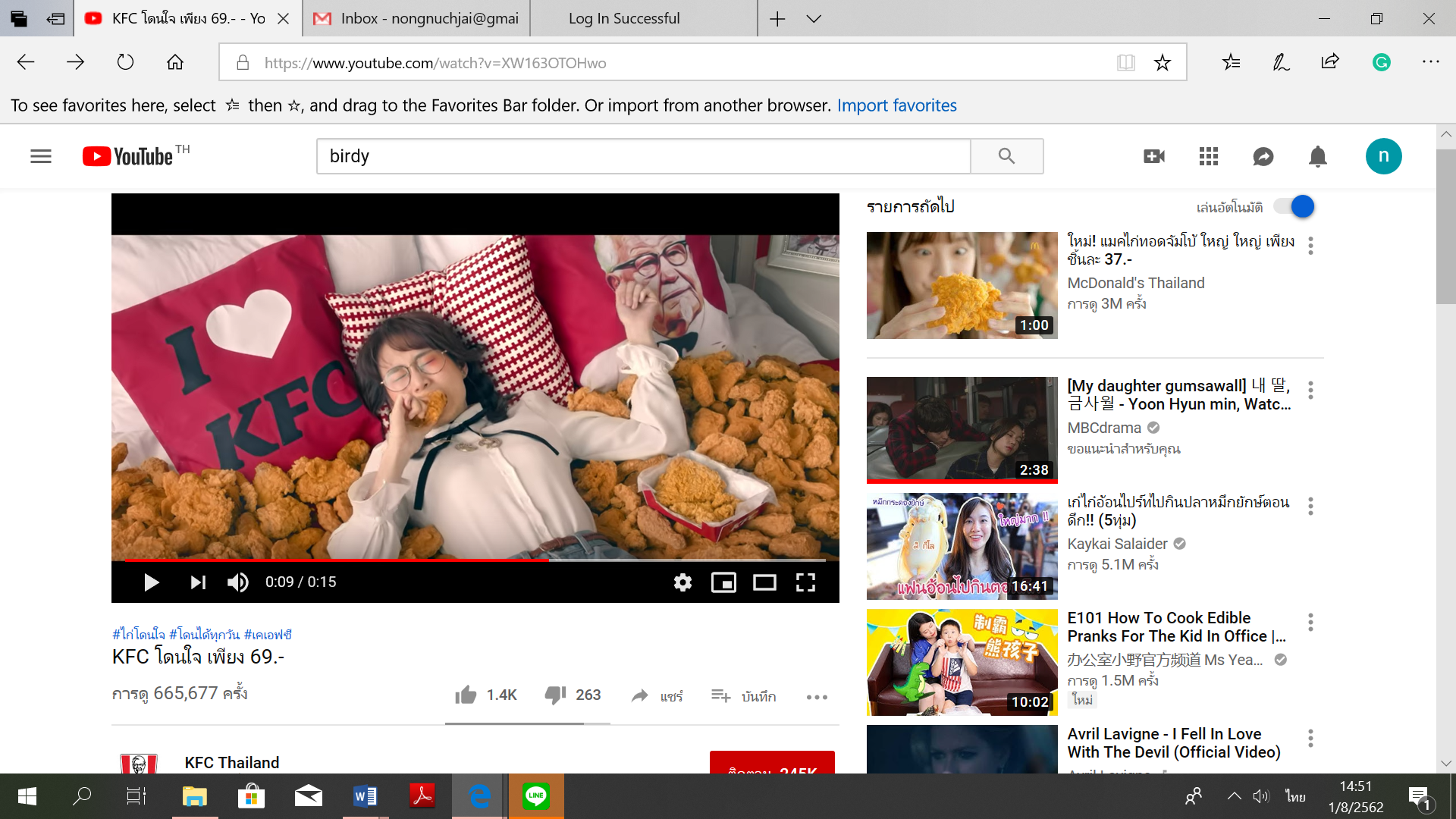 | Description:: KFC Don Jai |
| 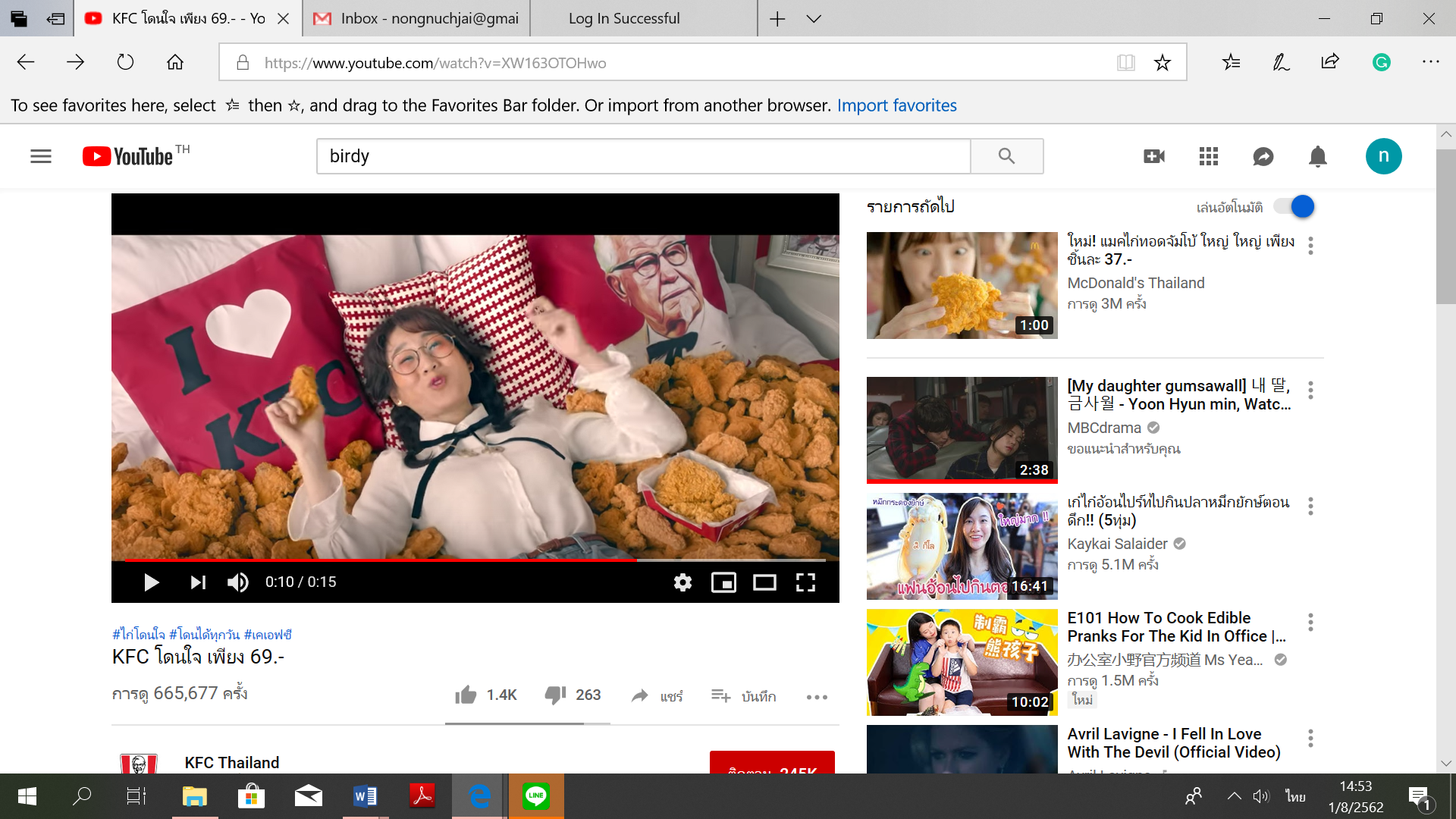 | Description:: Very Yummy |
| 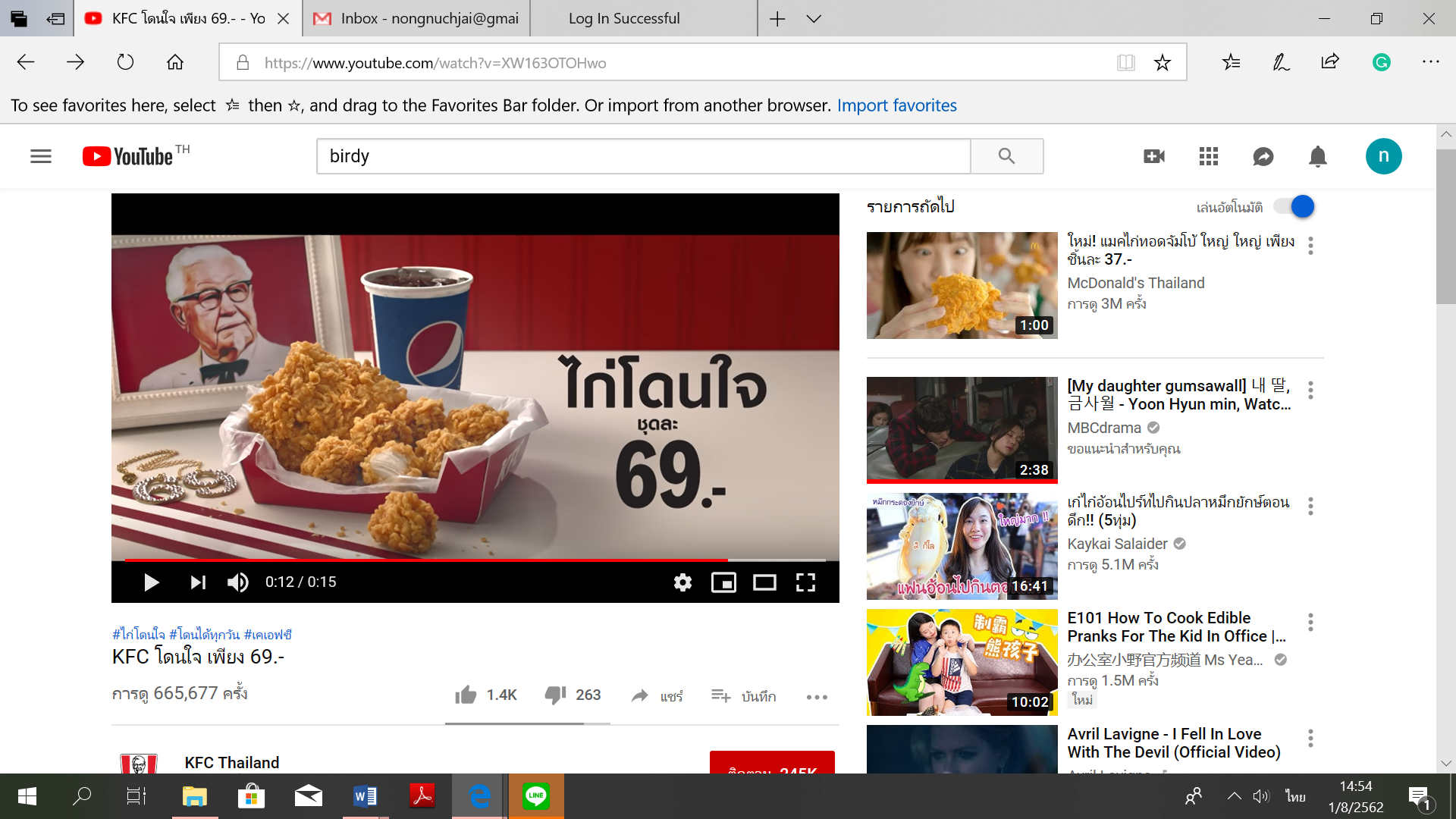 | Description:: Only 69 bahs |
| 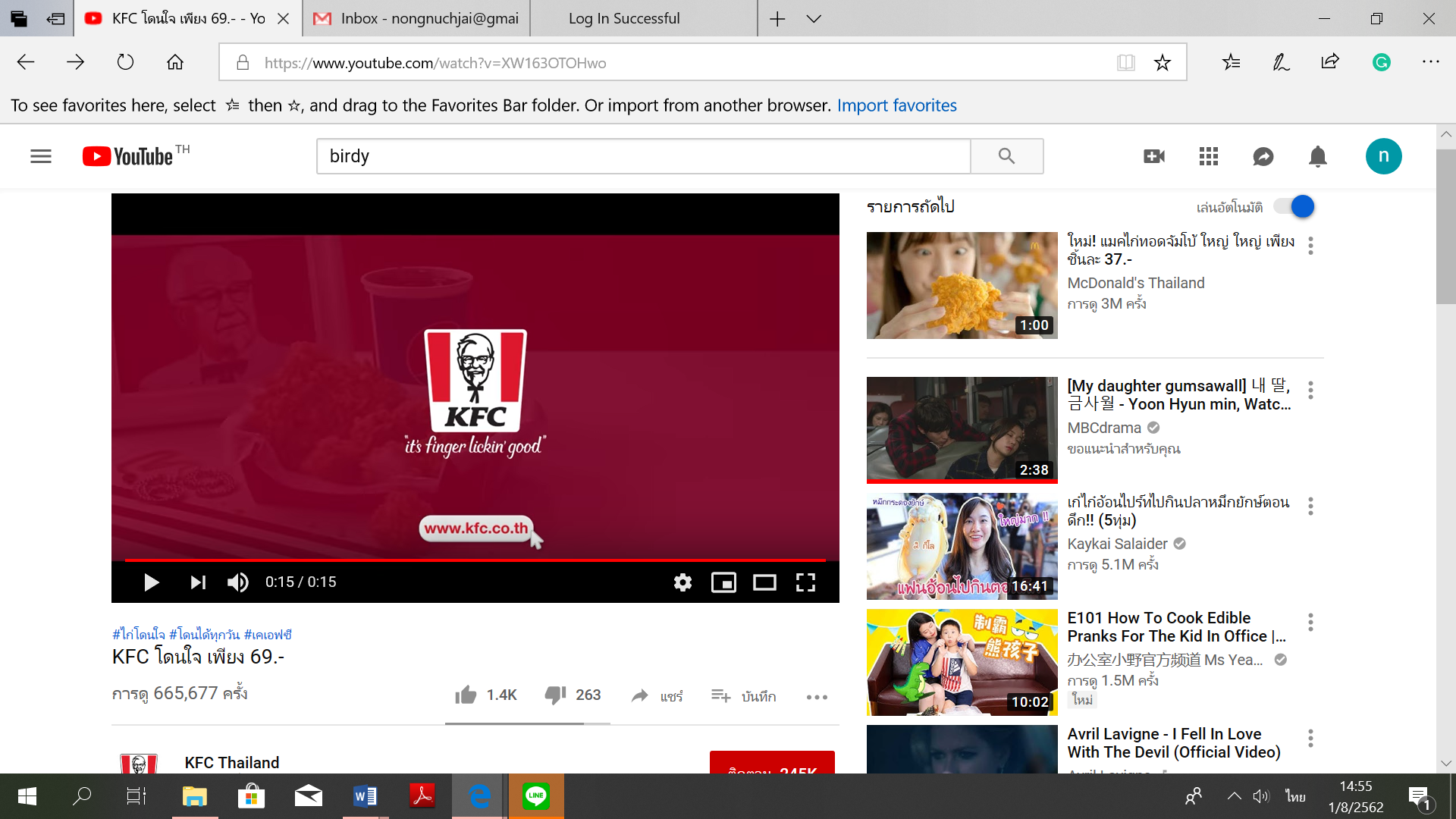 | Music |

**1. Do you know this clip?**

........1. No

........2. Yes

**2. Please answer these questions**

| **MHL** | **Question** | **Answer** | | | |
| --- | --- | --- | --- | --- | --- |
| **Perceive and understand** | 1. What product does this advertising sell? | Soft drink | Fried chicken | Do not sell any products | Mattress |
|  | 1. What content has appeared in the media that you just watched, to present a story about? | Special price of fried chicken | Eating fried chicken | Sleeping | Drinking soft drinks |
|  | 1. Is there the content about health appear in the media? | Yes | No |  |  |
| **Analyze** | 1. What would be the purpose of this advertisement? | To recommend new product | To show eating fried chhicken | To show sleeping on the beg | To show price of fried chicken |
|  | 1. Who are created this video clip? | Advertising agency | The company or product owner | Government agency | Both the company or product owner and advertising agency |
|  | 1. Where does the content and information from the presenter or the presenter in the advertisement come from? | True heart of the presenter ง | Dialogue written by the advertiser | Facts reflected by experts | All are correct. |
|  | 1. Who is target audience? | Children | Teengares | Adults | Everyone |
|  | 1. Who are get benefit from this video clip? | Advertisng agency | The company or product owner | Presenters | Audiences |
| **Evaluate** | 1. Do you like this video clip? | Yes | No |  |  |
|  | 1. Is this video clip reliable? | Yes | No |  |  |
|  | 1. Is this video clip benefit for you? | Yes | No |  |  |
| **Intent to take act** | 1. After watching this video clip, do you intend to buy or not? | Yes | No |  |  |
|  | 1. After watching this video clip, do you intend to tell your parents or guardians to buy or not? | Yes | No |  |  |
|  | 1. After watching this video clip, do you intend to tell your friends to buy or not? | Yes | No |  |  |
|  | 1. Will you take the information from this video clip to doin daily lifeor not, such as education, reporting or homework to send teachers? | Yes | No |  |  |

**Section 3 Health behviors**

**1. Eating**

1.1 Normally, how many meals a day do you eat?

........Eat more than 3 meals **(Skip to Q 1.3)**

........Eat 3 full meals **(skip to Q 1.3)**

........Eat 2 meals, skip breakfast

........Eat 2 meals, skip lunch

........Eat 2 meals, refrain from dinner

........Eat only 1 meal

1.2 Why did not you eat that meal?

........No money

........No time

........Lose weigh

........Observing

........Other (specify)......................................

1.3 During the past 1 week, how often you have the following food to eat?

| **Foods** | **Days/ week** | **Times/ day** |
| --- | --- | --- |
| 1. Snacks, sweet such as ice cream, chocolate candies, Thai desserts, cookies, cakes, wafers |  |  |
| 1. Sweetened beverages that add sugar, such as not 100% fruit juice or juices that add sugar, sweetened water, soft drinks |  |  |
| 1. Vegetable |  |  |
| 1. Fruit |  |  |

1.4 Do you do other activities while eating food?

| **Activities** | **Yes** | **No** |
| --- | --- | --- |
| 1. Watch TV |  |  |
| 1. Surf internet |  |  |
| 1. Play game |  |  |
| 1. Other (specify)………………………………….. |  |  |

1.5 Food literacy

Note: These questions is to access food literacy, particular knowledge and interactive skill and food choice domains**.**

**1.5.1 These statemets which are correct statements**

|  | Agree | Disgree |
| --- | --- | --- |
| **1.1 Food & nutrition knowledge** |  |  |
| - Eating salty foods such as crunchy snacks, fish, instant noodles are harmful to the body | 🗆 | 🗆 |
| - Drinking sweetened beverages or too much sugar are armful to the body | 🗆 | 🗆 |
| - Eating foods that are high in fat, such as sausages, fried chicken, can cause obesity. | 🗆 | 🗆 |
| - Reading the production and expiry dates food on the packaging before buying is important for health | 🗆 | 🗆 |

**1.5.2 Do you agree with these statements or not?**

|  | Agree | Disagree |
| --- | --- | --- |
| **2.1 Interactive skill domain** |  |  |
| - I can reject friends' invitations which encourages eating unhealthy food | 🗆 | 🗆 |
| - If I encounter eating unhealthy food at home or at school, I can tell people at home that I won't eat these foods | 🗆 | 🗆 |
| - If members of my family are fat and eating unhealthy food such as foods that are high in fat, I'll tell him to change his diet and eat healthy food | 🗆 | 🗆 |
| **2.2 Food choice** |  |  |
| - When I go shopping with my parents, I will buy food with a label which shows that "Good for health” | 🗆 | 🗆 |
| - I eat 5 food groups of food every day | 🗆 | 🗆 |

**2.** Physical activity literacy (PA literacy)

Note: These questions is to access PA literacy which focus onpsychosocial domain of level 1 (foundation & exploration) and 2 (acquisition & accumulation)

**1. Exercise behavior**

**1.1 Do you agree with these statements?**

|  | Yes / true / agree | No / not true / disagree |
| --- | --- | --- |
| - I feel excited and fun every time when I play sports games or doing activities that require physical movement. | 🗆 | 🗆 |
| - I don't like when there is anything to obstruct me playing sports or doing activities that require physical movement | 🗆 | 🗆 |
| - I feel shywhen I have to run, do physical activity, exercise, or play sports alone. | 🗆 | 🗆 |

**1.2 confidence**

|  | Yes / true / agree | No / not true / disagree |
| --- | --- | --- |
| - I like to try physical activities, exercise or play new sports. | 🗆 | 🗆 |
| - Running, playing, doing physical activities, exercising or playing sports make me more confident in my physical movements. | 🗆 | 🗆 |

**1.3 motivation**

|  | Yes / true / agree | No / not true / disagree |
| --- | --- | --- |
| - I have some motivation to make myself exercise or constantly moving | 🗆 | 🗆 |
| - I do exercise or doing activities that require physical movement only when someone tells I to do it. | 🗆 | 🗆 |
| - The goal is to exercise or doing activities that require physical movement. | 🗆 | 🗆 |

**2 Physical activity behaviors**

During the past 1 week

2.1 You have work activities that requires movement, exertion or use of physical strength so hard that makes breathing difficult heart rate beats up dramatically until feeling above breathless, never ending a sentence such as lifting or carrying heavy objects or digging the soil for at least 10 minutes or not?

........1. No

........2. Yes

Frequency

|  | **Day/ weeks** | **Timee/ day** | **minutes/ day** |
| --- | --- | --- | --- |
| weekday |  |  |  |
| weekend |  |  |  |

2.2 You have work activities that require movement, physical strength or use of moderate physical strength Which causes moderate breathing but is not suffocating, such as household chores, movement of items Planting trees, for example, for at least 10 minutes or not?

........1. No

........2. Yes

Frequency

|  | **Day/ weeks** | **Timee/ day** | **minutes/ day** |
| --- | --- | --- | --- |
| weekday |  |  |  |
| weekend |  |  |  |

**Section 4 Personal information**

Please answer these questions

1. Sex

........1. Male ........2. Female

1. Age...............years
2. What class are you currently studying in?

.........1. Did not study

.........2. Study

.........1. Grade 4

.........2. Grade 5

.........3. Grade 6

.........4. Grade 7

.........5. Grade 8

.........6. Grade 9

1. How much GPA did I get last term in last semeter?

..............................

**If being an elementary school student, use the grade criteria in this table.**

| **Grade** | **Rating range** | **Grade level** |
| --- | --- | --- |
| 4 | 80-100 | Excellent |
| 3.5 | 75-79 | Very good |
| 3 | 70-74 | good |
| 2.5 | 65-69 | Quite good |
| 2 | 60-64 | moderate |
| 1.5 | 55-59 | OK |
| 1 | 50-54 | Pass the minimum requirements |
| 0 | 0-49 | Below threshold |

1. Religion

……1. Buddha

……2. Islamic

……3. Christ

……4. Hindu

……5. Confucius

……6. No Religion

……7. Other (specify)……………………………………

1. Nationality

.........1. Thai .........2. Karen

.........3. China .........4. Myanmar

.........5. Laos .........6. Cambodia

.........7. Other (specify)............................................................

1. Shape

Weight..................kg.

Height...................cm.

1. In the past 1 year (or last year), have you ever been ill? (Have to take medicine to see a doctor)

........1. No (Skip to Q9)

........2. Yes

1. How often you are sick?

........1. More or equal 5 times per year

........2. Less ill or 4 times per year

1. Health status

........1. No congenital disease (healthy)

........2. Chronic diseases such as asthma, diabetes (chronic health problem)

1. Pocket money for buying food at school (approximately) ....................Bahts per day
2. Pocket money for buying sweets and beveragres (approximately) .................... Bahts per day

**Section 5 Family data**

1. Marital status of parents

.........1. Married and living together

.........2. Not married but living together

.........3. Widow

.........4. Divorce

.........5. Separated

.........6. Don’t know

1. Education of parents

**Father’s education**

.........1. Did not study

.........2. Primary education (Primary 1-6)

.........3. Lower secondary school (grade 1-3)

.........4. Upper Secondary (Secondary 4-6)

.........5. Vocational certification

.........6. Diploma / High Vocational certification

.........7. Bachelor degree

.........8. Master degree

.........9. Doctor degree

.........10. Other (specify)................................................................

**Mother’s education**

.........1. Did not study

.........2. Primary education (Primary 1-6)

.........3. Lower secondary school (grade 1-3)

.........4. Upper Secondary (Secondary 4-6)

.........5. Vocational certification

.........6. Diploma / High Vocational certification

.........7. Bachelor degree

.........8. Master degree

.........9. Doctor degree

.........10. Other (specify)................................................................

**Guardian’s education**

.........1. Did not study

.........2. Primary education (Primary 1-6)

.........3. Lower secondary school (grade 1-3)

.........4. Upper Secondary (Secondary 4-6)

.........5. Vocational certification

.........6. Diploma / High Vocational certification

.........7. Bachelor degree

.........8. Master degree

.........9. Doctor degree

.........10. Other (specify)................................................................

1. Occupation of parents

**Father’s occupation**

.........1. Civil servants such as teachers, police, soldiers

.........2. State enterprise employees

.........3. Private company employee

.........4. Government employee

.........5. Business owner

.........6. Agriculture

.........7. Fishing

.........8. General contractor

.........9. Housewife

.........10. Retire

.........11. Not working / Unemployed

.........12. Other (specify).............................................................

**Mother education**

.........1. Civil servants such as teachers, police, soldiers

.........2. State enterprise employees

.........3. Private company employee

.........4. Government employee

.........5. Business owner

.........6. Agriculture

.........7. Fishing

.........8. General contractor

.........9. Housewife

.........10. Retire

.........11. Not working / Unemployed

.........12. Other (specify).............................................................

**Guardian’s occupation**

.........1. Civil servants such as teachers, police, soldiers

.........2. State enterprise employees

.........3. Private company employee

.........4. Government employee

.........5. Business owner

.........6. Agriculture

.........7. Fishing

.........8. General contractor

.........9. Housewife

.........10. Retire

.........11. Not working / Unemployed

.........12. Other (specify).............................................................

1. Incomes of parents

Father’s incomes .................... Bahts per month

Mother’s incomes .................... Bahts per month

Guardian’s incomes .................... Bahts per month

**Section 6 Socio-economic data**

**1.** **Who are living with you?**  **(Answer more than 1 choice)**

.........1. Father and mother

.........2. Grandfather/ grandmother

.........3. Uncle/ aunt

.........4. Brother/ sister

.........5. Friend

.........6. Only father

.........7. Only mother

.........8. Other (Specify)........................................................

**2.** **Whom is the place of residence of the child or parents?**

.........1. Belonging to my own or my parents

.........2. Not belonging to my own or my parents, such as renting the guardian's workplace or living in other people's home

**3.** **What kind of residence of children and parents have?**

.........1. Single house

.........2. Condominium / suite / mansion

.........3. Townhouse / Twin house / Town home

.........4. Townhouse / Twin house / Town home

.........5. Flat / apartment / dormitory

.........6. Row house

.........7. Other (Specify)............................................................

**4. What kind of vehicles does the parent or family member have?** **(Answer more than 1 choice)**

…......1. Car

.........2. Motorbike

.........3. Bicycle

.........4. Wagon, tractor

.........5. Ship

.........6. Other (Specify)............................................................

**5. Do you have these applicances in your house? (Answer more than 1 choice)**

........1. Television

........2. Smart television

........3. Computer

........4. Laptop/netbook

........5. Tablet

........6. Telephone

........7. Mobile phone

........8. Smart phone, IOS system

........9. Smart phone, Android system

........10. Refrigerator

........11. Other (Specify)...............................................................

**######end of survey######**
