# Supplementary material for: Are exposure to health information and media health literacy associated with fruit and vegetable consumption?
Source: BMC Public Health. 2023 Aug 16;23:1554. doi: 10.1186/s12889-023-16474-1 (PMC10428547; doi:10.1186/s12889-023-16474-1)
Supplement: Supplementary file 2 — Additional file 2. [file 12889_2023_16474_MOESM2_ESM.docx]

*Table 1* *Fruit intake among Thai Children by* socio-demographic characteristics

| **Variables** | **Fruit intake** | | | |
| --- | --- | --- | --- | --- |
|  | **n** | **High**  **(5-7 days per week)** | **Moderate**  **(3-4 days per week)** | **Low**  **(1-2 days per week)** |
| **Overall** | **1,871** | **17.5** | **40.2** | **42.3** |
| Gender | | | | |
| Female | 1,035 | 18.7 | 42.8 | 38.5 |
| Male | 836 | 15.7 | 37.1 | 47.2 |
| Age | | | | |
| 10 | 336 | 15.5 | 40.5 | 44.0 |
| 11 | 405 | 17.8 | 36.9 | 45.4 |
| 12 | 455 | 18.7 | 38.7 | 42.6 |
| 13 | 371 | 19.7 | 42.6 | 37.7 |
| 14 | 304 | 14.1 | 44.1 | 41.8 |
| Place of residence | | | | |
| Rural | 945 | 16.5 | 39.0 | 44.4 |
| Urban | 926 | 18.4 | 41.4 | 40.2 |
| GPA | | | | |
| Low (1.00-2.99) | 586 | 13.0 | 37.2 | 49.9 |
| Moderate (3.00-3.50) | 781 | 17.2 | 39.6 | 43.3 |
| High (3.51-4.00) | 504 | 22.8 | 44.8 | 32.3 |
| Amount of pocket money | | | | |
| <20 baht | 706 | 15.2 | 40.7 | 44.2 |
| 20-50 baht | 1,110 | 18.7 | 40.3 | 41.0 |
| >50 baht | 55 | 18.2 | 34.5 | 47.3 |
| BMI (IOTF) | | | | |
| Underweight | 969 | 18.1 | 39.6 | 42.9 |
| Normal | 563 | 17.2 | 42.1 | 40.7 |
| Overweight/obese | 339 | 15.6 | 38.9 | 45.4 |
| Health status | | | | |
| Without a chronic illness | 1,688 | 17.8 | 40.7 | 41.6 |
| Having a chronic illness | 183 | 13.7 | 36.3 | 50.0 |

*Table 2* *Vegetable intake among Thai Children by* socio-demographic characteristics

| **Variables** | **Vegetable intake** | | | |
| --- | --- | --- | --- | --- |
|  | **n** | **High**  **(5-7 days per week)** | **Moderate**  **(3-4 days per week)** | **Low**  **(1-2 days per week)** |
| **Overall** | 1,871 | **40.3** | **35.0** | **24.7** |
| Gender | | | | |
| Female | 1,035 | 40.0 | 36.1 | 23.9 |
| Male | 836 | 40.8 | 33.7 | 25.5 |
| Age | | | | |
| 10 | 336 | 31.5 | 36.0 | 32.4 |
| 11 | 405 | 37.5 | 35.1 | 27.4 |
| 12 | 455 | 41/8 | 35.6 | 22.6 |
| 13 | 371 | 45.3 | 34.5 | 20.2 |
| 14 | 304 | 45.7 | 33.9 | 20.4 |
| Place of residence | | | | |
| Rural | 945 | 43.2 | 34.9 | 21.9 |
| Urban | 926 | 37.4 | 35.1 | 27.5 |
| GPA | | | | |
| Low (1.00-2.99) | 586 | 36.9 | 34.6 | 28.5 |
| Moderate (3.00-3.50) | 781 | 41.5 | 33.9 | 24.6 |
| High (3.51-4.00) | 504 | 42.7 | 37.3 | 20.0 |
| Amount of pocket money | | | | |
| <20 baht | 706 | 42.1 | 35.0 | 22.9 |
| 20-50 baht | 1,110 | 39.5 | 35.5 | 25.0 |
| >50 baht | 55 | 34.5 | 27.3 | 38.2 |
| BMI (IOTF) | | | | |
| Underweight | 969 | 37.4 | 35.0 | 27.7 |
| Normal | 563 | 40.3 | 36.2 | 23.4 |
| Overweight/obese | 339 | 49.0 | 33.3 | 17.7 |
| Health status | | | | |
| Without a chronic illness | 1,688 | 41.0 | 34.8 | 24.2 |
| Having a chronic illness | 183 | 34.1 | 37.9 | 28.0 |
